# Mapping and characterization QTLs for phenological traits in seven pedigree-connected peach families

**DOI:** 10.1186/s12864-021-07483-8

**Published:** 2021-03-16

**Authors:** Zena J. Rawandoozi, Timothy P. Hartmann, Silvia Carpenedo, Ksenija Gasic, Cassia da Silva Linge, Lichun Cai, Eric Van de Weg, David H. Byrne

**Affiliations:** 1 Department of Horticultural Sciences, Texas A&M University, College Station, TX 77843 USA; 2 Embrapa Clima Temperado, BR-392, km 78, Cx. Postal 403, Pelotas, Rio Grande do Sul 96010-971 Brazil; 3 Department of Agricultural and Environmental Sciences, College of Agriculture, Forestry and Life Sciences, Clemson University, Clemson, SC 29634 USA; 4 Department of Horticulture, Michigan State University, East Lansing, MI 48824 USA; 5 Plant Breeding, Wageningen University & Research, Wageningen, Netherlands

**Keywords:** FlexQTL, *Prunus persica* QTL, Haplotype, Pedigree-based analysis, Bloom date, Ripening date, Fruit development period

## Abstract

**Background:**

Environmental adaptation and expanding harvest seasons are primary goals of most peach [*Prunus persica* (L.) Batsch] breeding programs. Breeding perennial crops is a challenging task due to their long breeding cycles and large tree size. Pedigree-based analysis using pedigreed families followed by haplotype construction creates a platform for QTL and marker identification, validation, and the use of marker-assisted selection in breeding programs.

**Results:**

Phenotypic data of seven F_1_ low to medium chill full-sib families were collected over 2 years at two locations and genotyped using the 9 K SNP Illumina array. Three QTLs were discovered for bloom date (BD) and mapped on linkage group 1 (LG1) (172–182 cM), LG4 (48–54 cM), and LG7 (62–70 cM), explaining 17–54%, 11–55%, and 11–18% of the phenotypic variance, respectively. The QTL for ripening date (RD) and fruit development period (FDP) on LG4 was co-localized at the central part of LG4 (40–46 cM) and explained between 40 and 75% of the phenotypic variance. Haplotype analyses revealed SNP haplotypes and predictive SNP marker(s) associated with desired QTL alleles and the presence of multiple functional alleles with different effects for a single locus for RD and FDP.

**Conclusions:**

A multiple pedigree-linked families approach validated major QTLs for the three key phenological traits which were reported in previous studies across diverse materials, geographical distributions, and QTL mapping methods. Haplotype characterization of these genomic regions differentiates this study from the previous QTL studies. Our results will provide the peach breeder with the haplotypes for three BD QTLs and one RD/FDP QTL to create predictive DNA-based molecular marker tests to select parents and/or seedlings that have desired QTL alleles and cull unwanted genotypes in early seedling stages.

**Supplementary Information:**

The online version contains supplementary material available at 10.1186/s12864-021-07483-8.

## Background

Peaches and nectarines [*Prunus persica* (L.) Batsch] are deciduous fruit trees belonging to the Rosaceae family. These are native to China and grown throughout the world in a wide range of environments. The gross production value of peaches and nectarines in 2016 was $825 million in the United States and $17,107 million globally [1].

Breeding of woody perennial crops is not an easy task since their long juvenility periods and large plant size makes maintaining large populations in the field expensive [2]. The use of marker-assisted breeding (MAB) provides a tool to do an early selection of seedlings, identify superior parents, improve the selection of elite alleles for essential traits, and stack desirable alleles [3, 4]. This strategy is pertinent for perennial fruit tree to reduce breeding operational costs [3].

QTL identification in peaches conducted [5] for acidity, total sugar content, organic acids, fruit weight, bloom, and harvest dates [6, 7], and chilling injury susceptibility [8] have been limited due to the low marker density of genetic maps [9]. Recently, these issues have been overcome due to the availability of the peach genome v1.0 and v2.0 [10, 11] sequence and the development of the International Peach SNP Consortium peach 9 K SNP array [11]. Moreover,

the Pedigree-Based Analysis (PBA) approach [12, 13] that uses multiple pedigree-linked families allows the discovery of more QTL or QTL-alleles per locus across a range of genetic backgrounds. This approach has facilitated the identification of QTLs for blush [14–16], ripening date [15, 17, 18], soluble solids content [15–18], fruit weight, and titratable acidity [15, 17, 19].

Bloom date, which is primarily determined by chilling requirement [20–22], is an important trait determining peach adaptation for both low and high chill zones. Bloom date has been reported as moderately to a highly heritable trait (0.39–0.92) [15, 23–27]. QTLs for bloom date were reported on LG1 (40–60% of phenotypic variance (PVE)), LG2 (27% PVE), LG4 (32–35% PVE) and LG7 (21% PVE). Not all the QTLs were found in all the studies indicating the population-specific nature of these QTLs [15, 17, 28–30].

Ripening date in peach trees is a crucial element for extending the production season and developing cultivars that ripen throughout the harvest season. Also, the ripening process is involved in the regulation of several metabolic pathways such as blush, sugar/acid balance, and the flesh softening in peach fruits [31]. Narrow sense heritability (h^2^) for ripening date ranges from high to very high (0.79–0.94) [15, 32, 33]. The major QTL for controlling RD was mapped on LG 4 at ~ 44 cM in the *Prunus* T × E reference map, and a putative candidate gene was located at ~ 10.5 Mbp on the peach genome sequence v.1 [30, 31, 34, 35]. This QTL explained ~ 50 to 98% of the phenotypic variability. The RosBREED project has verified this locus is significant in the U.S. breeding programs [18]. Likewise, a QTL for RD on chromosome 4 was detected in apricot, sweet cherry [31], and almond [36].

Fruit development period (FDP) is the period between bloom and ripening dates [37], and is well correlated with RD [6, 15]. This trait is highly heritable (*h*^*2*^ = 0.73–0.98) [15, 23, 26, 38]. QTLs for fruit development period were mapped on LGs 1, 2, 3, 4, 5, and 6 with decisive evidence. The QTLs mapped by Hernández Mora, et al. [15] on LGs 1–6 and by Etienne, et al. [6] on LG4 co-localized with ripening date QTLs.

Currently, DNA-based tests for a few breeding-relevant traits have been developed and used in the peach marker-assisted selection application, including maturity date (G4mat) [39], quality traits, and fruit bacterial spot resistance. Thus, work is needed to develop DNA tests for BD and FDP traits and to validate SNP-based DNA test (G4mat) for ripening date to enable their use in the TX and other breeding programs [3, 40–42].

The objectives of this study are to identify new and/or validate the major QTLs previously reported for bloom date, ripening date, and fruit development period through pedigree-based analysis approach (PBA) using Texas peach/nectarine germplasm. Also, to estimate QTL genotypes for important breeding parents and to identify predictive SNP marker(s) associated with desired QTL alleles. Results from this research will facilitate the design of DNA tests linked to these QTL(s) or genes for routine use for marker-assisted breeding.

## Results

### Phenotypic data analysis

The mean BD value ranged from 42.3 ± 3.9 (CA11) to 50.2 ± 9.45 (TX13), and a maximum range of 51, with the number of observations between 82 in CA11 and 143 in overall mean (Additional file 1: Table S1). In our study, BD distribution varied across environments and overall mean (Additional file 2: Fig. S1). The CA environments were skewed towards the lower values, whereas the TX exhibited multimodal profiles with two or more peaks in both environments. This was expected as some of the higher chill genotypes had delayed bloom in the lower chill Texas site compared to California (~ 540 vs. ~ 1090 chilling hours) [43]. Normal distribution was seen in the overall mean of BD.

RD exhibited an average between 129.2 ± 16.7 (TX12) and 157.4 ± 17.7 (CA11), with greater (87.0) and lower (59.5) RD ranges in the overall mean and TX13 data sets, respectively. CA and the overall mean data sets were slightly skewed towards the higher values, while the TX data sets were skewed towards the lower values. On average, fruit ripened approximately 17 days later at Fowler, CA than at College Station, TX. FDP mean values ranged from 81.2 ± 16.9 (TX12) and 115.3 ± 16. 9 (CA11) with FDP range from ~ 67 (CA12) to 91 (TX13) days. The minimum number of observations (59) was recorded for CA11 compared to 138 observations for the CA12 and overall mean data sets. Similar to RD, FDP for CA data sets were slightly skewed towards higher values compared to the TX environments, which skewed towards lower values while the overall mean showed normal distribution. Fruit had development periods that were 23 days longer, on average, at Fowler, CA, than at College Station, TX. This was an effect of cooler temperatures during early fruit development in March and April for CA11 and CA12 (~ 15 and 9 °C) relative to TX12 and TX13 (21 and 18 °C).

Among these traits, a strong correlation was found between RD and FDP (r = 0.91) (Additional file 1: Table S2), and a moderately weak correlation was observed between FDP and BD (− 0.45). The negative correlations between BD and FDP suggest that the earlier blooming genotypes experience a delay in the rate of fruit development due to cooler temperatures. A weak correlation was found between RD and BD traits (− 0.14).

### Genotype by environment interactions

The genotype × environment interaction (G × E) is the differential sensitivity of genotypes to different environments. If such interaction exists, the selection would be complicated and result in genetic gains reduction in a breeding program. Understanding the G × E interactions is key to increasing the efficiency of marker-assisted selection for complex traits [44].

In this study, RD and FDP showed very high broad-sense heritability (H^2^ = 0.95 and 0.96, respectively), strong correlations among environments (r = 0.91), and minimal G × E variance (${\upsigma}_{\mathrm{g}\times \mathrm{e}}^2/{\upsigma}_{\mathrm{g}}^2$ ratio = 0.20) (Additional file 1: Table S3 and S4) whereas BD trait, showed highbroad-sense heritability (H^2^ = 0.88), strong correlation among environments (r = 0.83) and a moderate genotype by environment interaction (${\upsigma}_{\mathrm{g}\times \mathrm{e}}^2/{\upsigma}_{\mathrm{g}}^2=$ 0.70). All traits had comparable PC2 values and ranged from 5.5 to 6.8 (Additional file 1: Table S5), implying that the environments equally discriminate the populations for these traits. Finally, the minimal G × E effect of RD and FDP is supported by the relatively similar length of the environmental vectors in the GGE biplots, especially within the same location, indicating a high correlation among them and equal discriminatory ability of the four environments (Additional file 2: Fig. S2). Also, the distance between the environmental vectors was closer between CA11 and CA12, and between TX12 and TX13 for RD and FDP, respectively, illustrate that genotypes responded similarly in these two environments. This is confirmed by the highest positive correlations between CA11 and CA12 (r = 0.87, RD and 0.84, FDP) and between TX12 and TX13 (r = 0.79, RD and 0.89, FDP) for RD and FDP) (see Additional file 1: Table S4).

For BD, the sharper angle and less distance were observed between CA12 and TX12, TX12 and TX13, and CA12 with TX13 (Additional file 2: Fig. S2), indicating a stronger correlation between these environments (r = 0.73, 0.75, and 0.65) (Additional file 1: Table S4). The best discrimination of BD among genotypes was observed in the CA11 environment indicated by the longer vectors for these environments (Additional file 2: Fig. S2). Also, the environment CA11 was far from the other three environments and showed less correlation coefficient. However, the low number of observations of this environment (82) may have affected the correlation and G × E results.

### Genome-wide QTL analysis

The narrow-sense heritability (h^2^) varied among datasets in each trait. Minimum h^2^ (0.44) for BD was observed in BD-CA11 versus maximum observed h^2^ (0.82) in BD-mean (Table 1). While for RD, h^2^ ranged from 0.59 (RD-TX13) to 0.83 (RD-CA12), and for FDP, the minimal h^2^ was observed in FDP-CA11 (0.65) and the maximal in FDP-CA12 (0.82).

**Table 1:** QTLs mapped for the bloom date (BD), ripening date (RD), and fruit development period (FDP) traits evaluated in four environments (CA11, CA12, TX12, and TX13), and the overall mean for 143 peach seedlings

									***2ln(BF)***		
***Trait***	***MCMC***	***Records***	***μ***	***σ***^***2***^_***p***_	***σ***^***2***^_***e***_	***σ***^***2***^_***A***_	***h***^***2***^	***LG***	***1/0***	***2/1***	***3/2***
BD-CA11	150,000	82	42.3	15.2	8.5	6.7	0.44	1	6.6	0.1	0.0
BD-CA12	250,000	138	43.8	10.5	2.2	8.3	0.79	1	11.4	2.7	0.2
								4	10.4	0.3	−0.5
								7	29.5	1.0	−0.1
BD-TX12	150,000	114	49.3	76.3	23.5	52.9	0.69	1	5.1	1.3	0.7
								4	3.9	1.0	0.4
								7	15.6	1.3	0.6
BD-TX13	150,000	124	50.2	89.3	23.5	65.7	0.74	1	14.1	−0.4	−0.3
								4	29.6	−1.3	na
BD-mean	3600,000	143	47.0	42.6	7.6	35.1	0.82	1	13.9	5.5	−1.2
								4	4.6	−2.0	na
								7	14.6	−0.9	na
RD-CA11	100,000	104	157.4	313.9	97.6	216.3	0.69	4	28.0	3.9	0.6
RD-CA12	200,000	138	147.3	239.0	41.5	197.5	0.83	4	na	18.6	0.2
RD-TX12	100,000	94	129.2	278.8	112.6	166.1	0.60	4	29.3	0.6	−0.4
								7	2.3	0.2	na
RD-TX13	500,000	114	141.8	293.7	119.8	173.8	0.59	4	27.6	4.5	0.7
RD-mean	100,000	135	142.9	187.9	67.4	120.5	0.64	4	na	10.0	1.0
FDP-CA11	100,000	59	115.3	285.2	97.7	185.7	0.65	4	27.0	4.4	1.1
FDP-CA12	100,000	138	103.5	249.9	46.2	203.1	0.82	4	na	30.9	0.3
FDP-TX12	250,000	94	81.2	286.5	91.6	194.8	0.68	4	29.0	1.8	1.0
								6	4.5	1.3	0.0
FDP-TX13	150,000	114	91.3	321.0	105.5	215.4	0.67	4	28.2	3.6	1.0
FDP-mean	100,000	138	95.5	246.4	71.7	174.7	0.71	4	na	11.7	1.8

Bloom date, ripening date, and fruit development period in Julian days

*CA11* Fowler, California 2011, *CA12* Fowler, California 2012, *TX12* College Station, Texas 2012, *TX13* College Station, Texas 2013

Markov chain Monte Carlo (MCMC) run length, phenotypic mean (*μ*), phenotypic variance (*σ*^*2*^_*P*_), residual variance(*σ*^*2*^_*e*_), additive variance(*σ*^*2*^_*A*_), narrow-sense heritability (*h*^*2*^), the linkage groups (LG) that QTLs were mapped on

*2ln(BF)*. Bayes Factor, a measure quantifies the support from the data for the number of QTLs in the model (QTL evidence), after pair-wise model comparison (1/0, 2/1, and 3/2) such as ‘one-QTL model’ vs. ‘zero-QTL

Three QTLs were mapped for BD on three linkage groups (LG1, 4, and 7) across the four environments (site × year combinations) and their overall mean. The QTL on LG1 was at the distal end and showed strong to decisive evidence in all data sets (Table 1 and Additional file 2: Fig. S3). The QTL on LG4 was mapped in three environments (except CA11) and the overall analysis, showing positive and decisive evidence. At the same time, the QTL on LG7 was seen in only two environments and the overall analysis with decisive evidence. FlexQTL software found one to two candidate QTLs for RD and FDP depending on the environment; however, only the QTL on the middle part of G4 passed our inclusion criteria. (Table 1 and Additional file 2: Fig. S4 and S5).

**Fig. 1:**
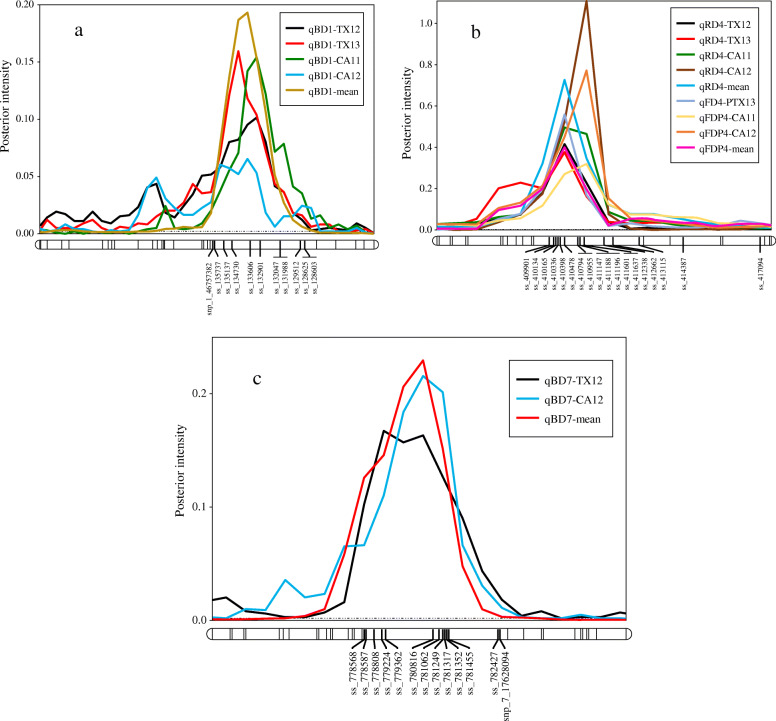
Position of putative QTLs and peaks controlling the bloom date (BD) in peach at linkage group 1 (LG1) **a** and LG7 **b** and the ripening date (RD) and fruit development period (FDP) at LG4 **c** from four environments (CA11, CA12, TX12, TX13), and the overall combined mean generated using MapChart software [45]. CA11, CA12 = Fowler, California 2011 and 2012; TX12, TX13 = College Station, Texas 2012 and 2013

For BD, the proportion of phenotypic variation explained (PVE) ranged from 17 to 54%, 11 to 55%, 11 to 18% for LG1, LG4, and LG7, respectively (Table 2). The highest posterior QTL intensity (0.96) showed in LG1 for BD-mean, and the lowest intensity (0.21) was found in LG4 for BD-TX12. The highest additive effect (~ 10 days) was in LG4 for BD-TX13, and the lowest (~ 2 days) showed in LG1, 4, and 7 for BD-CA12. The QTL on LG1 was co-localized across all data sets with an interval between 172 and 182 cM (peaks, 174, 176, and 178 cM), and the physical position of this chromosomal region was 43,058,300 - 45,586,061 bp on the peach genome sequence v2.0, (Table 2, Fig. 1a, and Additional file 1: Table S6). Likewise, peaks of QTL on LG4 of three data sets, except CA12, clustered at mode 50 cM, with an interval between 48 and 54 cM and physical chromosomal position between 11,956,738 – 13,633,831 bp. Regarding LG7, the peaks co-localized at either 64 or 66 cM with an interval from 62 to 70 cM and physical chromosomal position between 15,513,277 - 17,226,623 bp on the peach genome sequence v2.0 (Table 2 and Fig. 1b, Additional file 1: Table S6).

**Table 2:** QTL name, linkage group, interval, mode peak, intensity, additive effect, and phenotypic variance explained (PVE) for the bloom date (BD), ripening date (RD), and fruit development period (FDP) traits evaluated in four environments (CA11, CA12, TX12, and TX13), and the overall mean for 143 peach seedlings

***QTL name***	***Linkage Group***	***Interval (cM)***	***Mode peak (cM)***	***Intensity***	***Additive Effect (d)***	***PVE***
*qBD1*-CA11	1	[174, 182]	178	0.94	5	54
*qBD1*-CA12	1	[172, 180]	176	0.43	2	19
*qBD1*-TX12	1	[172, 182]	178	0.72	5	17
*qBD1*-TX13	1	[172, 182]	174	0.86	6	20
*qBD1*-mean	1	[172, 182]	178	0.96	5	35
*qBD4*-CA12	4	[70, 78]	76	0.60	2	18
*qBD4*-TX12	4	[48, 52]	50	0.21	4	11
*qBD4*-TX13	4	[48, 52]	50	0.85	10	55
*qBD4*-mean	4	[48, 54]	50	0.42	4	14
*qBD7*-CA12	7	[62, 70]	66	0.87	2	17
*qBD7*-TX12	7	[62, 70]	64	0.89	5	18
*qBD7*-mean	7	[62, 68]	66	0.91	3	11
*qRD4*-CA11	4	[42, 46]	44	1.40	17	46
*qRD4*-CA12	4	[42, 46]	45	1.80	19	75
*qRD4*-TX12	4	[42, 46]	44	0.85	18	54
*qRD4*-TX13	4	[40, 46]	44	1.21	17	52
*qRD4*-mean	4	[42, 46]	44	1.50	17	57
*qFDP4*-CA11	4	[42, 46]	45	1.10	16	42
*qFDP4*-CA12	4	[42, 46]	45	1.60	19	71
*qFDP4*-TX12	4	[46, 52]	50	0.79	18	56
*qFDP4*-TX13	4	[42, 46]	44	1.10	20	62
*qFDP4*-mean	4	[40, 46]	44	1.04	14	40

Bloom date, ripening date, and fruit development period in Julian days

*CA11* Fowler, California 2011, *CA12* Fowler, California 2012, *TX12* College Station, Texas 2012, *TX13* College Station, Texas 2013

Posterior intensity is the accumulated probability of QTL presence in a successive series of 2 cM bins (chromosome segments) based on Bayesian analysis

For each QTL reported, the evidence [*2ln(BF)*] is either positive (2–5), strong (5–10), or decisive (> 10)

The proportion of phenotypic variation explained by RD QTL on LG4 ranged between 46 and 75% (Table 2). The highest posterior QTL intensity (1.80) and the highest additive effect (~ 19 days) were found in CA12. In most environments, the observed high intensity (greater than one) implies that FlexQTL assigned two QTLs within the same QTL interval with an average distance between them of 1.0 cM across all sampled models. This distance is very short to be genetically meaningful for population sizes. This QTL had mode at either 44 or 45 cM, overlapping intervals from 40 to 46 cM across all data sets, and the physical chromosomal position between 10,396,616 to 11,298,736 on the peach genome sequence v2.0 (Table 2, Fig. 1c, and Additional file 1: Table S6). The proportion of phenotypic variation explained by FDP QTL on LG4 ranged between 40 and 71% (Table 2). The highest posterior QTL intensity (1.60) was for CA12 and the lowest (0.79) for TX12. The highest additive effect (~ 20 days) was found in TX13. Likewise, this QTL had a mode at either 44 or 45 cM, overlapping intervals from 40 to 46 cM across all data sets, except TX12, and has a physical chromosomal position between ~ 10,396,616 to 11,298,736 bp of the peach genome sequence v2.0 (Table 2 and Additional file 1: Table S6). Like RD, the high intensity that is noticed in most data sets indicates two tightly linked QTLs within the QTL interval, and the gap between them averaged to 1.4 cM across all sampled models. So, the distance is also too short to be genetically dissected in these studied population sizes.

### QTL associated haplotypes, number of QTL-alleles, their effect, predictive markers, and sources

On LG1, 11 SNPs in the predicted *qBD*G1 region (172.23–182.34 cM) (Additional file 1: Table S7), chosen for haplotyping, revealed eight SNP haplotypes across the seven parents in which H8 was a common haplotype (Table 3). The estimation of the diplotype effect identified families of two parents (Y434–40 and ‘Victor’) were segregating for this QTL. The results also discovered multiple *Q*-alleles of various effects associated with H1 to H7, and only one *q*-allele was linked to low phenotypic values associated with H8.

**Table 3:** QTL genotypes for bloom date (BD), ripening date (RD), and fruit development period (FDP) traits for seven breeding parents, with associated linkage groups, haplotype names, the haplotype’s SNP sequences, and original sources

Trait/LG/Pos	Parents	QTL allele	Hap.	SNP haplotype	Successive ancestors
				Allele sequence	(founders in bold)
BD LG1 [172.23–182.34]	Galaxy	*Q ♀*	H4	**AB**ABBBBBAAB	**Galaxy**
	Galaxy	*Q ♂*	H4	**AB**ABBBBBAAB	**Galaxy**
	Y426–371	*Q*_*1*_ *♀*	H1	**AB**ABBBAAAAB	**Y426–371**
	Y426–371	*Q*_*1*_ *♂*	H7	**BA**BBBBAAABB	**Y426–371**
	**Y434–40**	*Q*_*4*_ *♂*	H2	**AB**ABBBAAABB	**Y434–40**
	**Victor**	*Q ♂*	H5	**AB**BBBBABBBA	Goldprince > **F_Goldprince**
	Y435–246	*Q*_*3*_ *♀*	H6	**BA**ABBBBBAAB	**Y435–246**
	Y435–246	*Q*_*2*_ *♂*	H3	**AB**ABBBABBBA	**Y435–246**
	**Y434–40**	*q ♀*	H8	**BB**ABBBBB**AAA**	**Y434–40**
	**Victor**	*q ♀*	H8	**BB**ABBBBB**AAA**	TropicBeauty > **Fla3–2**
	TX2B136	*q ♀*	H8	**BB**ABBBBB**AAA**	**TX2B136**
	TX2B136	*q ♂*	H8	**BB**ABBBBB**AAA**	**TX2B136**
	TXW1490_1	*q ♀*	H8	**BB**ABBBBB**AAA**	TropicBeauty > **Fla3–2**
	TXW1490_1	*q ♂*	H8	**BB**ABBBBB**AAA**	**F_TXW1490_1**
BD LG4 [47.83–54.54]	TX2B136	*Q ♀*	H3	AB**A**A**A**AB**BA**ABAB	**TX2B136**
	TX2B136	*Q ♂*	H3	AB**A**A**A**AB**BA**ABAB	**TX2B136**
	TXW1490_1	*Q ♀*	H3	AB**A**A**A**AB**BA**ABAB	TropicBeauty > **Flordaprince**
	TXW1490_1	*Q ♂*	H3	AB**A**A**A**AB**BA**ABAB	**F_TXW1490_1**
	**Y426–371**	*Q ♀*	H3	AB**A**A**A**AB**BA**ABAB	**Y426–371**
	**Victor**	*Q ♂*	H3	AB**A**A**A**AB**BA**ABAB	Goldprince > **F_Goldprince**
	**Y435–246**	*Q ♂*	H2	B**A**BBBB**A**ABBA**B**A	**Y435–246**
	**Galaxy**	*Q ♂*	H2	B**A**BBBB**A**ABBA**B**A	**Galaxy**
	**Y435–246**	*q ♀*	H1	B**BB**BBBBABAAAA	**Y435–246**
	**Y426–371**	*q ♂*	H1	B**BB**BBBBABAAAA	**Y426–371**
	**Galaxy**	*q ♀*	H1	B**BB**BBBBABAAAA	**Galaxy**
	Y434–40	*q ♂*	H1	B**BB**BBBBABAAAA	**Y434–40**
	Y434–40	*q ♀*	H4	A**BB**BBBBABAAAA	**Y434–40**
	**Victor**	*q ♀*	H5	A**BB**ABABABBBAB	TropicBeauty > **Fla3–2**
BD LG7 [62.05–68.91]	Y435–246	*Q ♂*	H3	AB**A**AABAABBABB	**Y435–246**
	**Galaxy**	*Q ♀*	H6	BB**A**BABBABABBA	**Galaxy**
	**Victor**	*Q ♂*	H6	BB**A**BABBABABBA	Goldprince > **F_Goldprince**
	**TX2B136**	*Q ♀*	H1	BB**A**BBAAAABAAB	**TX2B136**
	**Y426–371**	*Q ♂*	H2	BB**A**BBAAAABABA	**Y426–371**
	Y435–246	*Q ♀*	H2	BB**A**BBAAAABABA	**Y435–246**
	**Y434–40**	*Q ♀*	H2	BB**A**BBAAAABABA	**Y434–40**
	**Galaxy**	*q ♂*	H4	AA**B**ABBBBBABAB	**Galaxy**
	**Y426–371**	*q ♀*	H4	AA**B**ABBBBBABAB	**Y426–371**
	**Y434–40**	*q ♂*	H5	AA**B**AAAAAABABA	**Y434–40**
	**Victor**	*q ♀*	H7	AA**B**BBAAAABABA	TropicBeauty > **Flordaprince**
	**TX2B136**	*q ♂*	H7	AA**B**BBAAAABABA	**TX2B136**
	TXW1490_1	*q ♀*	H7	AA**B**BBAAAABABA	TropicBeauty > **Flordaprince**
	TXW1490_1	*q ♂*	H7	AA**B**BBAAAABABA	**F_TXW1490_1**
RD and FDP LG4 [42.33–45.19]	**Y426–371**	*Q*_*1*_ *♂*	H3	B**AA**A**A**A**AA**AB**A**AAA**B**	**Y426–371**
	**Y434–40**	*Q*_*1*_ *♂*	H3	B**AA**A**A**A**AA**AB**A**AAA**B**	**Y434–40**
	**Galaxy**	*Q*_*1*_ *♀*	H3	B**AA**A**A**A**AA**AB**A**AAA**B**	**Galaxy**
	**Victor**	*Q*_*2*_ *♀*	H4	A**AA**B**A**B**AA**BA**A**BBB**B**	TropicBeauty > **Fla3–2**
	**TXW1490_1**	*Q*_*2*_ *♀*	H4	A**AA**B**A**B**AA**BA**A**BBB**B**	TropicBeauty > **Fla3–2**
	Y435–246	*q*_*1*_ *♀*	H1	A**BB**B**B**B**BB**BA**B**BBB**A**	**Y435–246**
	Y435–246	*q*_*1*_ *♂*	H1	A**BB**B**B**B**BB**BA**B**BBB**A**	**Y435–246**
	**Y434–40**	*q*_*1*_ *♀*	H1	A**BB**B**B**B**BB**BA**B**BBB**A**	**Y434–40**
	**Galaxy**	*q*_*1*_ *♂*	H1	A**BB**B**B**B**BB**BA**B**BBB**A**	**Galaxy**
	**Victor**	*q*_*1*_ *♂*	H1	A**BB**B**B**B**BB**BA**B**BBB**A**	Goldprince > **F_Goldprince**
	TX2B136	*q*_*1*_ *♀*	H1	A**BB**B**B**B**BB**BA**B**BBB**A**	**TX2B136**
	TX2B136	*q*_*1*_ *♂*	H1	A**BB**B**B**B**BB**BA**B**BBB**A**	**TX2B136**
	**TXW1490_1**	*q*_*1*_ *♂*	H1	A**BB**B**B**B**BB**BA**B**BBB**A**	**F_TXW1490_1**
	**Y426–371**	*q*_*2*_ *♀*	H2	B**BB**B**B**B**BB**BA**B**BBB**A**	**Y426–371**

QTL alleles for each parent cultivar are presented with ♀ and ♂ for maternal and paternal parent sources, respectively. Parents that are heterozygous for the QTL are in bold. Allele(s) for predictive SNP marker(s) associated with *Q* or *q*-alleles for increasing or decreasing a given trait, respectively, are shown in**underscored bold**. *Q*/*q* of different effect magnitude are indicated by subscript numbers. The identity of the SNP markers and their physical and genetic location is given in Additional file 1: Table S7

**Fig. 2:**
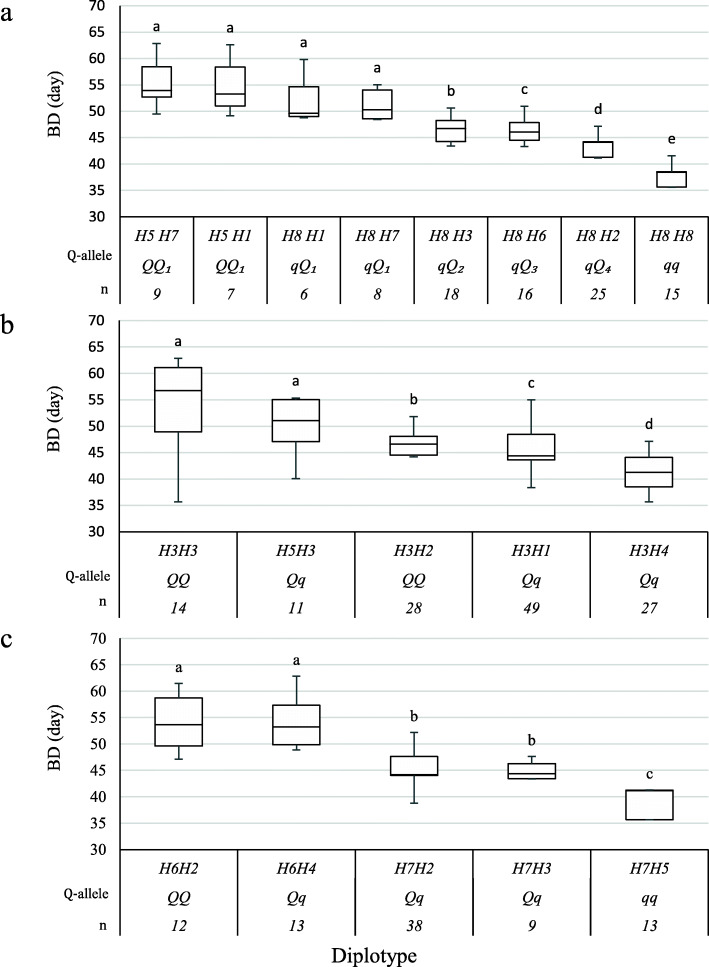
Diplotype effect of the most common haplotypes associated with bloom date (BD) for the three QTLs mapped on LG1 **a**, LG4 **b**, and LG7 **c**. Means not connected by the same letter are significantly different (*P < 0.05*) within each linkage group. *n* = Diplotype sample size

The examination of the haplotype /diplotype effects (Fig. 2a) revealed that the effect of H7 and H1could not differentiated when comparing H5H7<>H5H1 and H8H1<>H8H7. Likewise, the effects of H5 and H8 could not be differentiated when comparing H5H1 to H8H1 and H5H7 to H8H7. Also, H7 had a larger effect than H8 and H3 in the comparison H8H7<>H8H8 and H8H7<>H8H3, respectively. The effect size of H1 was greater than H2 and H3 when comparing H8H1 to H8H2 and H8H3. In general, H8 had a smaller effect than H1, H2, H3, H6, and H7, when comparing H8H8 to H8H1, H8H2, H8H3, H8H6, and H8H7. Hence, H1 and H7 had similar and the largest effects, and both coined as *Q*1, then followed by H3, H6, H2, and H8, which were represented as *Q*2, *Q*3, *Q*4, and *q*, respectively. However, the under-representation of QTL genotypes hindered the estimation of H4 and H5 effects.

All of these haplotypes could be differentiated from H8 by various pairs of adjacent SNP markers by contrasting either *AB*- or *BA*-alleles for 1) snp_1_46757382 and ss_135737 to *BB* of H8, or 2) ss_128625 and ss_128603 to *AA* of H8, and 3) ss_129512 and ss_128603 to also *AA* of H8 (Table 3 and Additional file 1: Table S7). Breeding parents ‘Galaxy’, Y426–371, Y435–246, Y434–40, and TX2B136 were considered as founders in this study and the sources of these SNPs were unknown because their ancestors were not available for genotyping. On the other hand, the *Q-*allele (H5) of ‘Victor’ was inherited from F_Goldprince, and the *q-*allele (H8) of both ‘Victor’ and TXW1490–1 was inherited from Fla3–2 through ‘TropicBeauty’.

On LG4, there were 13 SNP markers in the BD QTL region (47.83 to 54.54 cM) (Additional file 1: Table S7) selected for haplotyping. That revealed five SNP haplotypes in the seven parents. H1 and H3 were the most common haplotypes (Table 3). Families of four parents (Y435–246, Y426–371, ‘Galaxy’, and ‘Victor’) were heterozygous for this QTL. H2 and H3 were associated with the *Q-*allele while H1, H4, and H5 with the *q-*allele.

The examination of the haplotype/diplotype effects in Fig. 2b revealed that H3 was not different from H5 based on H3H3<>H5H3. Also, H3 had a larger effect than H1, H2, and H4 when comparing H3H3 to H3H1, H3H2, and H3H4, respectively. Our results suggest different effects/magnitudes of some haplotypes on BD, e.g., H5H3 (*qQ*) had a larger effect than H3H2 (*QQ*). That could be explained by several reasons such as the presence of interaction with other loci, H5 *(q*) having a smaller effect on decreasing BD among the other haplotypes (H1 and H4) associated with decreasing BD, or H2 (*Q*) having less magnitude on increasing BD. The low number of diplotype observations or high variance within a diplotype class might also have caused these issues.

More than one predictive SNP marker associated with H2 and H3 (*Q*- allele) were identified (Table 3). A-allele at ss_415301 (50.09 cM) along with three more SNP markers distinguished H3, whereas the A-allele at ss_414387 (48.43 cM) and the other two SNP markers were unique for H2. In contrast, H1, H4, and H5 (*q-*allele) were distinguished by two adjacent BB-alleles at ss_414387 and ss_415301. The H3 *Q*- allele was found in TX2B136, ‘Flordaprince’, F_TXW1490_1, Y426–371, and F-Goldprince while the H2 *Q*- allele came from Y435–246 and ‘Galaxy’. The *q*- alleles were found in Y435–246, Y426–371, ‘Galaxy’, Y434–40, Fla3–2, and ‘TropicBeauty’.

On LG7, the 13 SNPs (62.05–68.91 cM) in the BD QTL region (Additional file 1: Table S7) were chosen for haplotyping. Seven SNP haplotypes were discovered across the seven parents (Table 3). Estimation of the diplotype effect found families of five parents (Y426–371, Y434–40, ‘Victor’, ‘Galaxy’, and TX2B136) were segregating for this QTL. H1, H2, H3, and H6 were assigned to the *Q-*allele and H4, H5, and H7 to *q-*allele (Table 3). The analysis of the haplotype/effects showed that the effects of H2 and H4 could not be differentiated based on H6H2<>H6H4, and the same was observed between H2 and H3 when comparing H7H2 <>H7H3 (Fig. 2c). H6 had a greater effect than H7 in the comparison H6H2 to H7H2. While H5 showed a smaller effect than H2 and H3 when comparing H7H5 to both H7H2 and H7H3, respectively. Likewise, the different effects of haplotypes were noticed in this QTL for the same reasons mentioned earlier. The A-allele at the SNP marker ss_778808 (15.6 Mb, 62.48 cM) (Table 3) was associated with *Q-*alleles. This SNP allele inherited from the parents ‘Galaxy’, Y426–371, Y435–246, Y434–40, and TX2B136. The sources of *q*- allele came from F_TXW1490_1, ‘Galaxy’, Y426–371, Y434–40, TX2B136, and from ‘Flordaprince’ through ‘TropicBeauty’.

15 SNP markers in the predictive QTL region for both RD and FDP traits (42.33 to 45.19 cM) (Additional file 1: table S7), on the middle part of LG4, were picked for haplotype analyses. FlexQTL implies this genomic region had more than one QTL within the same interval

Results discovered four SNP haplotypes associated with RD and FDP across the seven parents of which H1 was common (Table 3). Families of five parents (Y426–371, Y434–40, ‘Galaxy’, ‘Victor’, and TXW1490–1) were segregating in this region.

**Fig. 3:**
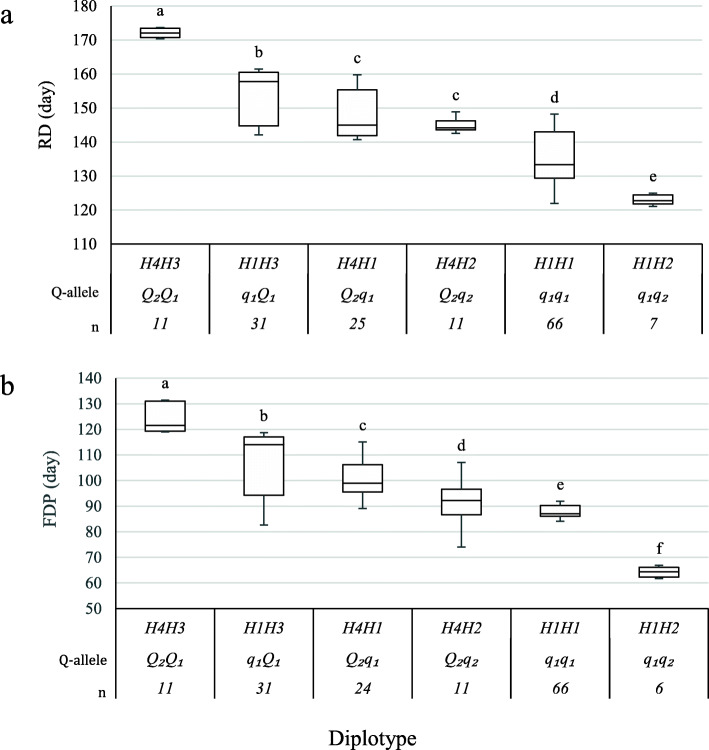
Diplotype effect of the most common haplotypes associated with ripening date (RD) **a** and fruit development period (FDP) **b** for the QTLs mapped on LG4. Means not connected by the same letter are significantly different (*P < 0.05*) within each linkage group. *n* = Diplotype sample size

The diplotype analysis revealed the presence of four statistically distinct phenotypic classes (Fig. 3 a and b). H3 had a larger effect than H1 and H4 when comparing H4H3<>H4H1 and H1H3 <> H4H1, respectively. Likewise, H2 showed a smaller effect than H1 on both RD and FDP when comparing H1H1<>H1H2 and from H4H1<>H4H2 just in FDP not RD as their effects could not be differentiated (Fig. 3 a and b). Thus, the effect size of haplotypes can be ordered as H3 > H4 > H1 > H2 that is differentiated by *Q*1, *Q2*, *q1*, and *q2*, respectively. The major finding in this study was the presence of multiple QTL alleles of different effects for a single locus. That may explain why the Bayes Factor values and high intensities of most data sets of this study suggested the presence of two QTLs.

Seven SNP markers were identified, each of which distinguished H3 and H4 from the other two haplotypes (Table 3). In this study, ‘Galaxy’, TX2B136, Y426–371, Y435–246, and Y434–40, were considered founders as their direct parents, and earlier generations do not exist or were not available for genotyping. *Q1* (H3) was found in parent Y426–371, Y434–40, and ‘Galaxy’ (Table 3), while *Q2* (H4) inherited from Fla3–2 through ‘TropicBeauty’. *q1* (H1) was from Y434–40, Y435–246, ‘Galaxy’, TX2B136, F_Goldprince, and F_TXW1490–1. Y426–371 parent was the only source of *q2* (H2). Thus, RD and FDP shared the same specific haplotypes and favorable SNP alleles associated with increasing/decreasing phenotypic values.

## Discussion

In this study, the bloom date was moderate to highly heritable (0.44–0.82) as has been previously reported [15, 24–27] in a range of germplasm, indicating that expression of bloom date is not heavily influenced by environmental effects which were supported by G × E results. Narrow sense heritability was moderate to high for RD (0.59 to 0.83) as was found in previous studies [15, 18, 26, 46–48]. FDP also has an important additive genetic component as indicated by a high to very high (0.65 to 0.82) estimated narrow-sense heritability reported in this and previous studies [15, 24–27].

Our QTL for BD on LG1 was flanked by snp_1_46757382 and ss_128603, spanned the region from 43.1–45.6 Mb with PVE from ~ 17 to 54%. This QTL was previously described in different germplasm, by Romeu, et al. [30] in the ‘V6’ × ‘Granada’ progeny (low- medium chill) (41.2 Mb) at the end of LG1, PVE ~ 60%) and by Fan, et al. [29] using ‘Contender’ (high chill) and ‘Fla.92-2C’ (low chill) population (at 45.6 Mb, PVE ~ 40%).

The QTL at the middle region of LG4 for BD mapped between ss_413934 and ss_419614, in the interval between 12 and 13.6 Mb, and PVE ranged from 11 to 55%. This QTL overlaps with the BD QTL on LG4 (qFD4.2) at nearest markers ss_417840 and ss_440116 (13.1 to 16.0 Mb) reported by Hernández Mora, et al. [15].

Lastly, the QTL at the distal end of LG7 was flanked by ss778568 and snp_7_17628094, spanned from 15.5 to 17.2 Mb and explained ~ 11 to 18% of BD phenotypic variation. This finding agreed with Romeu, et al. [30] who found a QTL for BD on LG7 at the nearest marker ss_779224 (15.7 Mb), which was close to our QTL peaks (ss_780816 (16.3 Mb) and ss_779362 (15.7 Mb)). Moreover, this region overlapped with the QTL (15.4 to 19.4 Mb; PVE ~ 60%) reported by Fan, et al. [29].

The only one of the three QTLs was detected in CA11 is probably due to that this environment had a low number of phenotypic data (82 records). The G × E for BD in the studied populations may result from the response of the high-chill seedlings to the lack of chill hours that delayed the blooming period.

In summary, this study provides more evidence that three mapped QTLs for BD on LG1, 4, and 7 are major loci for controlling BD and were supported by other studies using low- and medium-chill germplasm and bi-parental family mapping. It was also supported by the polygenic nature of BD inheritance. Additional QTLs for BD were also reported on LG2 [15, 49], LG3 [17, 30], LG6 [15, 30, 50], and LG8 [15, 17]. Thus, further studies using more diverse germplasm will be important to continue to characterize additional QTLs and candidate genes to identify the genetic pathway regulating the BD in peach.

The examination of haplotype/diplotype effects uncovered the high prevalence of a few haplotypes, e.g., H8 (*q*-allele), H3 (*Q*-allele), and H7 (*q*-allele) on LG1, 4, and 7, respectively, reflecting the relatively narrow genetic base of peach germplasm. Also, the results revealed the presence of multiple *Q*-alleles of different effects for the QTL on LG1 (*Q1, Q2, Q3,* and *Q4*) along with only one *q*-allele. In general, the small family sizes and consequently the low/lack representation of various compound diplotypes (e.g., 6 to 9 observations in some diplotypes of LG1) hindered the ability to make conclusions on the haplotype effects (H4 and H5) or the interplay among the three mapped QTLs for BD**.**

One QTL associated with RD and FDP was mapped at the middle part of LG4 (10.4–11.3 Mb) with PVE 46–75% and 40–71%, respectively. This specific genomic region was reported as associated with RD trait previously by Nuñez-Lillo, et al. [35] (~ 10.9 Mb), Romeu, et al. [30] (~ 10.7 Mb), Frett [18] (10.7–11.3 Mb), Eduardo et al. (2011; 2013) (~ 11.0–11.2 Mb) with candidate gene ppa008301m for maturity, and Hernández Mora, et al. [15] (~ 11.2–14.1 Mb). This held true using early-, mid-, and late-maturing populations. The co-localization between QTLs for RD and FDP was supported by the strong correlation (r = 0.87) (data not shown) between these traits in this study as well as previous work [6, 15].

Also, all data sets, except TX12, showed decisive evidence (BF ≥ 10) with high intensity for the presence of a second QTL on LG4. This could be explained by that TX12 had higher temperatures during the critical fruit development months (March and April) [51] compared to other sites. The higher temperatures accelerated RD and shortened FDP in this environment, which minimized the phenotypic variation as mentioned earlier (Additional file 1: Table S1).

Furthermore, the haplotype analysis of this chromosomal region revealed multiple predictive loci (ss_410398, ss_410794, and ss_412662) for decreasing and increasing for either RD or FDP. Likewise, examining the relative effects of haplotypes and estimated QTL genotypes revealed a series of QTL alleles of different effect at this locus that we coined *Q*1, *Q*2, *q*1, and *q*2. The use of multi-parent populations for finding multiple functional alleles of different effect was also reported for two acidity QTLs/genes in apple by Verma, et al. [52] and for the blush QTL in peach using the current germplasm by Rawandoozi, et al. [16]. In our germplasm, the RD QTL on LG4 co-localized with a QTL for soluble solids concentration (SSC) and blush reported by Rawandoozi, et al. [16]. These co-localizations had also been reported by other studies [15, 34, 53]. A pleiotropic effect of the RD has been reported on several quality traits [15, 34, 35, 39]. Co-factor analysis could be useful in future studies to account for one trait when analyzing another, e.g., accounting for RD for analyzing SSC or blush traits.

Overall, additional QTL mapping through pedigree-based analysis across a wider range of breeding germplasm is needed to identify and characterize additional QTLs to understand the whole genetic pathway controlling RD and FDP traits. Moreover, larger family sizes would ensure better representation of QTL genotype classes for estimating QTL effects and allow improved downstream analysis in case of multiple QTL alleles of different effects at a single locus and/or gene by gene interaction.

At the genomic region of the detected QTLs for these traits, candidate genes have been reported. For BD, the QTL interval (43,058,300 - 45,586,061 bp) of LG 1, the most promising candidate genes for the major QTL affecting blooming time and chilling requirement in LG1 were the Dormancy-associated MADS-box (DAM) genes within the evergrowing (*evg*) locus in peach, apricot, and almond [29, 54, 55].

Prupe.1G531600 (DAM5) and Prupe.1G531700 (DAM6) genes were identified as potential candidate genes of lateral bud endodormancy release in peach [29, 56, 57]. Prupe.1G531500 gene is described as MADS-box protein short vegetative phase (SVP) and it plays a role in controlling meristem development during the vegetative phase and flower development as well as in floral meristem determination [58]. Prupe.1G549600 and Prupe.1G548000 genes are described as agamous-like MADS-box proteins AGL11 and AGL12, respectively. AGL11 is a vital gene to control ovule identity and associated placental tissues in Arabidopsis [59]. While a MADS-box gene AGL12 regulates root development and flowering transition in Arabidopsis [60]. Prupe.1G554100 (AGL80) is also a member of the MADS-box family of genes. In Arabidopsis, AGL80 was found to be involved in female gametophyte development [61].

Likewise, many candidate genes have been reported within the interval (11,956,738-13,633,831 bp) of LG4. Prupe.4G208000 is described as a Forkhead-associated (FHA) domain-containing protein (DDL) that plays an important role in plant growth and development [62]. Prupe.4G197000 gene was proposed to link to auxin synthesis and response which is known to be involved in fruit set and ripening [63]. Prupe.4G202200, Fertilization Independent Endosperm (FIE) polycomb group protein, in *Arabidopsis thaliana* FIE regulates endosperm and embryo development and suppresses flowering during embryo and seedling development [64]. Prupe.4G207300 (uclacyanin) is associated with pollen grain development in rice [65]. Prupe.4G205500 (early nodulin-like protein 1) gene is reported to be engaged in determining the reproductive potential in *Arabidopsis* [66]. In the QTL region (15,513,277-17,226,623 bp) of LG7, Prupe.7G130900, CURLY LEAF (CLF) gene, is associated with the repression of FLOWERING LOCUS T (FT) gene and other flowering-time genes during the vegetative growth of the plant [67]. Prupe.7G153400 gene is described as a ATP-dependent DNA helicase (DDM1), the importance of this gene was previously reported for DNA methylation in genes and transposable elements [68]. Prupe.7G133100 (Zeaxanthin epoxidase) gene has been identified to play an important role in resistance to stresses, seed development, and dormancy in *Arabidopsis* [69].

Within the RD/FDP locus on LG4 (10,582,092 to 11,298,736), a list of candidate genes has been previously reported in this region. NAC072 (Prupe.4G816800) is the candidate gene for controlling the ripening date in peach [39]. Also, there are three other genes proposed to be involved in the determination of RD/FDP in peach. Prupe.4G79900 gene is needed for normal embryo development in *Arabidopsis* and maize [70, 71]. Prupe.4G179800 gene is described as Early nodulin- like protein 1 and PtNIP1in *Arabidopsis* and loblolly pine, respectively [72]. It is expressed in immature zygotic and somatic embryos of developing seeds. Prupe.4G179200 gene with functional annotation Purine permease 10 in *Arabidopsis* and OsPUP7 in rice [73], and showed a flowering delay in rice. Finally, Prupe.4G185800 [74] and Prupe.4G187100 [75] genes that were reported to be associated with the regulation of the anthocyanin biosynthetic pathway in peach. Hence, these results confirming the pleiotropic effect of the RD on several quality traits, including blush that was previously reported [15, 16, 34, 35, 39].

## Conclusions

The pedigree-based analysis was successfully used as a statistical method for discovering and validating QTLs. Four QTLs associated with three important phenological traits were validated using low- medium-chill peach/nectarine germplasm. Two minor QTLs were also identified. This approach increases the genetic background explored, improves statistical power, and allows the simultaneous detection and validation of QTLs.

QTLs for BD on LG1, 4, and 7 were verified, and the SNP haplotypes associated with increasing or decreasing BD were identified. A single QTL with multiple QTL alleles of different effects was detected on the central part of LG4 for both RD and FDP. Our findings would help breeding programs make crossing decisions to pick the combination of parents that have SNP haplotypes associated with lowering BD to produce progeny with better adaptation to subtropical environments like Texas or increasing BD to ensure better adaptation to temperate environments, whereas the results of RD and FDP will facilitate better targeting for specific ripening periods. Ultimately, the SNP haplotypes associated with these QTLs could be converted into easy-to-use high throughput markers (e.g., Simple Sequence Repeat (SSR), Kompetitive Allele-Specific PCR (KASP), and Sequence Characterized Amplified Region (SCAR) markers) to routinely use in MAB. In general, this approach would save time and resources, particularly for fruit breeders since perennial wood species have long juvenility periods, and large populations are expensive to maintain in the field.

## Methods

### Plant materials

Briefly, we included in this study 143 seedlings from seven related F_1_ families derived from seven parents descending from 12 founders. The parents are all cultivated germplasm that has been developed by the Stone Fruit Breeding programs at Texas A&M University in College Station, TX and the USDA Stone Fruit Breeding program in Parlier, CA (36° 36′ 25.19″ N; − 119° 31′ 22.19″ W). TX2B136, ‘Victor’, TXW1490_1 from the Texas germplasm are mainly derived from ‘Tropic Beauty’ and related selections of Florida peach germplasm and ‘Goldprince’ and ‘Springbrite’ developed in the USDA Stone Fruit Breeding program in Byron, GA. On the other hand, ‘Galaxy’, Y435–246, Y424–40, and Y426–371 were developed by the USDA Stone Fruit Breeding program from ‘Armking’ and germplasm from Rutgers University (New Brunswick, NJ), the University of Florida (Gainesville, FL), and Georgia USDA Stone Fruit Breeding program (Byron, GA). Seedlings and parental genotypes were grown in College Station, TX (30°37′41.60″N, 96°22′27.38″W), and Fowler, CA (36°38′21.37″N, 119°42′20.51″W). Full details on plant materials and plot establishment and design can be found in Rawandoozi, et al. [16].

### Phenotypic evaluations

Phenotypic data were taken at both locations across 2 years (2011–2012 in CA, and 2012–2013 in TX) on individual trees for three phenological traits, bloom date (BD), ripening date (RD), and fruit development period (FDP). The date of first (10% blossoms open) and full bloom (60 to 80% of the blossoms open) were visually assessed in the field and recorded for each tree. Ripening date was determined when 20% of fruits are pickable by visually inspecting the presence of a few soft fruits in the field for maturity two times per week. Both full bloom and ripening dates were converted to Julian days (0–365). FDA is difference in days between BD and RD.

### Heritability and G × E

Variance components for the studied traits were estimated using a linear mixed model with the residual maximum likelihood (REML). Results from REML were used to estimate the broad-sense heritability across the environments, as explained by Rawandoozi, et al. [16]. The R package GGEBiplots version 0.1.1 was used to estimate the variations due to genotypes and G × E. Pearson’s correlation coefficients were also estimated among phenotypic traits within and across the environments using R software version 4.0.3.

### Genotyping and linkage map

Plant samples were genotyped using the IPSC 9 K SNP Array for Peach [11], and SNP data were curated following the workflow described by Vanderzande, et al. [76]. After filtration, a total of 1487 informative SNPs were distributed over eight chromosomes using a conversion factor in which every 1 Mb corresponded to 4 cM [76].

### QTL mapping

FlexQTL software (version 0.1.0.42) with an additive genetic model conducted by Markov Chain Monte Carlo (MCMC) simulation was used for QTL mapping. The analysis was run at least three times on each data set. Different prior and maximum QTL numbers were used in each run to reach effective chain size (ECS) ≥ 100 for the mean, variance of the error, number of QTLs, and QTL variance, as recommended to draw reliable and accurate conclusions [13, 77]. MCMC length ranged from 100,000 to 3600,000 iterations to store one thousand samples with a thinning between 100 and 3600. Convergence was evaluated visually via trace and intensity plots [13]. Twice the natural logarithm of Bayes Factors [2ln(BF)] obtained from FlexQTL software used as evidence for presence and number of QTLs [78]. The 2ln(BF) value greater than 2, 5, or 10 indicate positive, strong, and decisive evidence, respectively. In this study, loci were considered if QTL had 2lnBF ≥ 5 or that 2 ≤ 2lnBF < 5 for at least two data sets, the QTLs with overlapping intervals of at least 2 cM on the same linkage group, and explained at least 10% of the phenotypic variation.

The additive (${\sigma}_{A(trt)}^2\Big)$, phenotypic $\left({\sigma}_P^2\right)$, and residual $\left({\sigma}_e^2\right)$ variances were obtained from FlexQTL output to estimate the narrow-sense heritability (h^2^), and the proportion of phenotypic variance explained (PVE) as follows:

$$ {h}^2=\frac{\sigma_{A(trt)}^2}{\sigma_P^2}\times 100\ \mathrm{where}:{\sigma}_{A(trt)}^2\ \mathrm{is}\ \mathrm{the}\ \mathrm{variance}\ \mathrm{of}\ \mathrm{the}\ \mathrm{trait} $$

$$ PVE=\frac{\sigma_{A(qtl)}^2}{\sigma_P^2}\times 100\mathrm{where}:{\sigma}_{A(qtl)}^2\mathrm{isthevarianceofQTL} $$

The QTL nomenclature in this study described by Rawandoozi, et al. [16] is a modification of that of Fan et al. [29].

### Haplotypes analysis

SNPs within the significant QTL interval were considered for haplotype analysis using the FlexQTL software and PediHaplotyper package of R [19]. Haplotype effects were determined from combinations of diplotypes by comparing the effects of the H1|H2 and H1|H3 diplotypes. The nonparametric multiple comparison Steele–Dwass test (*P* < 0.05) was used to assess the significance of differences using JMP Pro Version 13.2 (SAS Institute Inc., Cary, NC, 2016) as described by Rawandoozi, et al. [16].

## Supplementary Information

**Additional file 1; Supplemental Tables S1-S7**

**Additional file 2: Supplemental Figure S1-S5.**

## Data Availability

The genotypic and phenotypic datasets of seven full-sib peach families used in this study can be found in the Dryad Repository, 10.5061/dryad.tmpg4f4vp (https://datadryad.org/stash/share/oWBiP7isZFdQbY8zS0nTubqrhrT0RntovILSNJp9Xxc).

## Abbreviations

BF: Bayes factor
CG: Candidate gene
cM: Centimorgan
DNA: Deoxyribonucleic acid
ECS: Effective chain size
F_1_: First filial generation
FS: Full-sib
h^2^: Narrow-sense heritability
H^2^: Broad-sense heritability
LG: Linkage group
Mb: Megabase pair
MCMC: Markov Chain Monte Carlo
PVE: Phenotypic variance explained
QTL: Quantitative trait loci
SNP: Single nucleotide polymorphism
BD: Bloom date
RD: Ripening date
FDP: Fruit development period
MAB: Marker-assisted breeding
