# Supplementary material for: Mapping and characterization QTLs for phenological traits in seven pedigree-connected peach families
Source: BMC Genomics. 2021 Mar 16;22:187. doi: 10.1186/s12864-021-07483-8 (PMC7962356; doi:10.1186/s12864-021-07483-8)
Supplement: Supplementary file 1 — Additional file 1; Supplemental Tables S1-S7 [file 12864_2021_7483_MOESM1_ESM.docx]

| Table S1. Mean, minimum, maximum, standard deviation, and number of observations (N) for bloom date (BD), ripe date (RD), and fruit development period (FDP) in different environments. | | | | | | |
| --- | --- | --- | --- | --- | --- | --- |
| Trait | Environment | Mean | Min | Max | SD | N |
| BD | CA11 | 42.3 | 36.0 | 57.0 | 3.90 | 82 |
|  | CA12 | 43.8 | 37.0 | 51.0 | 3.24 | 138 |
|  | TX12 | 49.3 | 29.0 | 70.0 | 8.74 | 114 |
|  | TX13 | 50.2 | 29.0 | 80.0 | 9.45 | 124 |
|  | Overall mean | 47.0 | 33.5 | 67.0 | 6.53 | 143 |
|  | | | | | | |
| RD | CA11 | 157.4 | 131.0 | 204.0 | 17.72 | 104 |
|  | CA12 | 147.3 | 125.0 | 187.0 | 15.46 | 138 |
|  | TX12 | 129.2 | 101.0 | 185.0 | 16.70 | 94 |
|  | TX13 | 141.8 | 113.0 | 200.0 | 17.14 | 114 |
|  | Overall mean | 142.9 | 120.0 | 179.5 | 13.71 | 138 |
|  | | | | | | |
| FDP | CA11 | 115.3 | 88.0 | 156.0 | 16.89 | 59 |
|  | CA12 | 103.5 | 78.0 | 145.0 | 15.81 | 138 |
|  | TX12 | 81.2 | 53.0 | 127.0 | 16.93 | 94 |
|  | TX13 | 91.3 | 52.0 | 143.0 | 17.91 | 114 |
|  | Overall mean | 95.5 | 62.3 | 140.0 | 15.70 | 138 |
| CA11, CA12 = Fowler, California 2011 and 2012; TX12, TX13 = College Station, Texas 2012 and 2013; Overall mean = mean across environments for the trait.  BD = bloom date in Julian days; RD = ripe date in Julian days; FDP = fruit development period in Julian days. | | | | | | |

| Table S2. Pairwise correlations among bloom date (BD), ripe date (RD), and fruit development period (FDP) of seven full-sib families of peach. | | | |
| --- | --- | --- | --- |
| Trait | by Trait | Correlation coefficient (r) | Number of observations |
| BD | RD | -0.14 ^**^ | 356 |
| BD | FDP | -0.45 ^**^ | 355 |
| RD | FDP | 0.91 ^**^ | 388 |
| ^**^ Pearson correlation is significant at *P≤* *0.0*1 (2-tailed).  BD = bloom date in Julian days; RD = ripe date in Julian days; FDP = fruit development period in Julian days. | | | |

| Table S3. Estimates of variance components for genotype ($\sigma_{g}^{2}$), genotype × environment ($\sigma_{g\times e}^{2}$), and the ratio of ${\sigma_{g\times e}^{2}}/{\sigma_{g}^{2}}$ and broad-sense heritability (H^2^) for bloom date (BD), ripe date (RD), and fruit development period (FDP) of seven full-sib families of peach. | | | | |
| --- | --- | --- | --- | --- |
| Trait | $\sigma_{g}^{2}$ | $\sigma_{g\times e}^{2}$ | ${\sigma_{g\times e}^{2}}/{\sigma_{g}^{2}}$ | H^2^ |
| BD | 28.99 | 20.23 | 0.70 | 0.88 |
| RD | 258.13 | 52.88 | 0.20 | 0.95 |
| FDP | 280.13 | 57.15 | 0.20 | 0.96 |
| BD = bloom date in Julian days; RD = ripe date in Julian days; FDP = fruit development period in Julian days. | | | | |

| Table S4. Pairwise correlations among four environments for bloom date (BD), ripe date (RD), and fruit development period (FDP) of seven full-sib families of peach. | | | | |
| --- | --- | --- | --- | --- |
| Trait | Environment | by Environment | Correlation coefficient (r) | Number of observations |
| BD | CA11 | CA12 | 0.43 ^**^ | 66 |
|  | CA11 | TX12 | 0.34 ^*^ | 49 |
|  | CA11 | TX13 | 0.44 ^**^ | 57 |
|  | CA12 | TX12 | 0.73 ^**^ | 104 |
|  | CA12 | TX13 | 0.65 ^**^ | 103 |
|  | TX12 | TX13 | 0.75 ^**^ | 98 |
|  | Among environments | | 0.83 ^**^ |  |
| RD | CA11 | CA12 | 0.87 ^**^ | 59 |
|  | CA11 | TX12 | 0.56 ^**^ | 32 |
|  | CA11 | TX13 | 0.67 ^**^ | 43 |
|  | CA12 | TX12 | 0.66 ^**^ | 84 |
|  | CA12 | TX13 | 0.67 ^**^ | 102 |
|  | TX12 | TX13 | 0.79 ^**^ | 79 |
|  | Among environments | | 0.91 ^**^ |  |
| FDP | CA11 | CA12 | 0.84 ^**^ | 104 |
|  | CA11 | TX12 | 0.61 ^**^ | 62 |
|  | CA11 | TX13 | 0.61 ^**^ | 79 |
|  | CA12 | TX12 | 0.69 ^**^ | 85 |
|  | CA12 | TX13 | 0.68 ^**^ | 105 |
|  | TX12 | TX13 | 0.89 ^**^ | 82 |
|  | Among environments | | 0.91 ^**^ |  |
| ^*, **^ Pearson correlation is significant at *P≤* *0.0*5 and *P≤* *0.0*1 (2-tailed), respectively.  CA11, CA12 = Fowler, California 2011 and 2012; TX12, TX13 = College Station, Texas 2012 and 2013.  BD = bloom date in Julian days; RD = ripe date in Julian days; FDP = fruit development period in Julian days. | | | | |

| Table S5. Trait-wise principal component 1 and 2 variances (PC1 and PC2) of total GGE variation in BD, RD, and FDP evaluated at Fowler, CA 2011 and 2012 and College Station, TX in 2012 and 2013 environments of seven full-sib families of peach. | | | |
| --- | --- | --- | --- |
| Traits | GGE | | |
|  | PC1 | PC2 | Sum |
| BD | 87.9 | 6.8 | 94.7 |
| RD | 89.2 | 6.4 | 95.6 |
| FDP | 90.1 | 5.5 | 95.6 |
| BD = bloom date in Julian days; RD = ripe date in Julian days; FDP = fruit development period in Julian days. | | | |

| Table S6. QTL name, linkage group, along with SNP name, genetic position, and physical location of flanking markers and nearest marker to the center of the mode for the bloom date (BD), ripening date (RD), and fruit development period (FDP) traits evaluated in four environments (CA11, CA12, TX12, and TX13), and the overall combined mean for 143 peach seedlings. | | | | | | | | | | | | |
| --- | --- | --- | --- | --- | --- | --- | --- | --- | --- | --- | --- | --- |
|  | | | ***Flanking markers*** | | | | | | ***Nearest marker*** | | | |
| ***QTL name*** | ***Linkage***  ***group*** | | | ***Name*** | ***Genetic***  ***position (cM)*** | | | ***Physical***  ***location*** | ***Name*** | ***Genetic***  ***position (cM)*** | | ***Physical***  ***location*** |
| *qBD1*-CA11 | | 1 | ss_134730 | | | 174.31 | 43,578,596 | | ss_132901 | | 177.40 | 44,350,825 |
|  | |  | ss_128603 | | | 182.34 | 45,586,061 | |  | |  |  |
| *qBD1*-CA12 | | 1 | snp_1_46757382 | | | 172.23 | 43,058,300 | | ss_133606 | | 176.28 | 44,069,867 |
|  | |  | ss_131988 | | | 179.65 | 44,913,729 | |  | |  |  |
| *qBD1*-TX12 | | 1 | snp_1_46757382 | | | 172.23 | 43,058,300 | | ss_132901 | | 177.40 | 44,350,825 |
|  | |  | ss_128603 | | | 182.34 | 45,586,061 | |  | |  |  |
| *qBD1*-TX13 | | 1 | snp_1_46757382 | | | 172.23 | 43,058,300 | | ss_134730 | | 174.31 | 43,578,596 |
|  | |  | ss_128603 | | | 182.34 | 45,586,061 | |  | |  |  |
| *qBD1*-mean | | 1 | snp_1_46757382 | | | 172.23 | 43,058,300 | | ss_132901 | | 177.40 | 44,350,825 |
|  | |  | ss_128603 | | | 182.34 | 45,586,061 | |  | |  |  |
| *qBD4*-CA12 | | 4 | ss_446745 | | | 70.35 | 17,587,156 | | ss_465473 | | 75.38 | 18,845,078 |
|  | |  | ss_469044 | | | 76.83 | 19,206,580 | |  | |  |  |
| *qBD4*-TX12 | | 4 | ss_413934 | | | 47.83 | 11,956,738 | | ss_415301 | | 50.09 | 12,523,245 |
|  | |  | ss_417094 | | | 51.89 | 12,971,285 | |  | |  |  |
| *qBD4*-TX13 | | 4 | ss_413934 | | | 47.83 | 11,956,738 | | ss_415301 | | 50.09 | 12,523,245 |
|  | |  | ss_417094 | | | 51.89 | 12,971,285 | |  | |  |  |
| *qBD4*-mean | | 4 | ss_413934 | | | 47.83 | 11,956,738 | | ss_415301 | | 50.09 | 12,523,245 |
|  | |  | ss_419614 | | | 54.54 | 13,633,831 | |  | |  |  |
| *qBD7*-CA12 | | 7 | ss_778568 | | | 62.05 | 15,513,277 | | ss_780816 | | 65.46 | 16,365,104 |
|  | |  | snp_7_17628094 | | | 68.91 | 17,226,623 | |  | |  |  |
| *qBD7*-TX12 | | 7 | ss_778568 | | | 62.05 | 15,513,277 | | ss_779362 | | 63.14 | 15,784,304 |
|  | |  | snp_7_17628094 | | | 68.91 | 17,226,623 | |  | |  |  |

| Table S6. *Cont.* | | | | | | | |
| --- | --- | --- | --- | --- | --- | --- | --- |
| *qBD7*-mean | 7 | ss_778568 | 62.05 | 15,513,277 | ss_780816 | 65.46 | 16,365,104 |
|  |  | ss_781455 | 66.27 | 16,567,648 |  |  |  |
| *qRD4*-CA11 | 4 | ss_409901 | 42.33 | 10,582,092 | ss_410794 | 43.56 | 10,890,653 |
|  |  | ss_412662 | 45.19 | 11,298,736 |  |  |  |
| *qRD4*-CA12 | 4 | ss_409901 | 42.33 | 10,582,092 | ss_411601 | 43.91 | 10,976,364 |
|  |  | ss_412662 | 45.19 | 11,298,736 |  |  |  |
| *qRD4*-TX12 | 4 | ss_409901 | 42.33 | 10,582,092 | ss_410794 | 43.56 | 10,890,653 |
|  |  | ss_412662 | 45.19 | 11,298,736 |  |  |  |
| *qRD4*-TX13 | 4 | ss_409453 | 41.59 | 10,396,616 | ss_410794 | 43.56 | 10,890,653 |
|  |  | ss_412662 | 45.19 | 11,298,736 |  |  |  |
| *qRD4*-mean | 4 | ss_409901 | 42.33 | 10,582,092 | ss_410794 | 43.56 | 10,890,653 |
|  |  | ss_412662 | 45.19 | 11,298,736 |  |  |  |
| *qFDP4*-CA11 | 4 | ss_409901 | 42.33 | 10,582,092 | ss_411601 | 43.91 | 10,976,364 |
|  |  | ss_412662 | 45.19 | 11,298,736 |  |  |  |
| *qFDP4*-CA12 | 4 | ss_409901 | 42.33 | 10,582,092 | ss_411601 | 43.91 | 10,976,364 |
|  |  | ss_412662 | 45.19 | 11,298,736 |  |  |  |
| *qFDP4*-TX12 | 4 | ss_413115 | 46.38 | 11,593,768 | ss_415301 | 50.09 | 12,523,245 |
|  |  | ss_417094 | 51.89 | 12,971,285 |  |  |  |
| *qFDP4*-TX13 | 4 | ss_409901 | 42.33 | 10,582,092 | ss_410794 | 43.56 | 10,890,653 |
|  |  | ss_412662 | 45.19 | 11,298,736 |  |  |  |
| *qFDP4*-mean | 4 | ss_409901 | 42.33 | 10,582,092 | ss_410794 | 43.56 | 10,890,653 |
|  |  | ss_412662 | 45.19 | 11,298,736 |  |  |  |
| For each QTL reported, the evidence [*2ln(BF)*] is either positive (2-5), strong (5-10) or decisive (>10). | | | | | | | |

| Table S7. QTL name, linkage group, along with SNP name, genetic position, and physical location of flanking markers and nearest marker to the center of the mode for the for the bloom date (BD), ripening date (RD), and fruit development period (FDP) traits evaluated in four environments, and the overall mean for 143 peach seedlings. | | | | | | | | | | |
| --- | --- | --- | --- | --- | --- | --- | --- | --- | --- | --- |
| **SNP name** | **Genetic position (cM)** | **Physical location** | **Haplotype** | | | | | | | |
| ***qBD1*** | | | **H1** | **H2** | **H3** | **H4** | **H5** | **H6** | **H7** | **H8** |
| snp_1_46757382 | 172.23 | 43,058,300 | A | A | A | A | A | B | B | B |
| ss_135737 | 172.44 | 43,109,980 | B | B | B | B | B | A | A | B |
| ss_135137 | 173.35 | 43,337,658 | A | A | A | A | B | A | B | A |
| ss_134730 | 174.31 | 43,578,596 | B | B | B | B | B | B | B | B |
| ss_133606 | 176.28 | 44,069,867 | B | B | B | B | B | B | B | B |
| ss_132901 | 177.40 | 44,350,825 | B | B | B | B | B | B | B | B |
| ss_132047 | 179.62 | 44,904,968 | A | A | A | B | A | B | A | B |
| ss_131988 | 179.65 | 44,913,729 | A | A | B | B | B | B | A | B |
| ss_129512 | 181.79 | 45,448,596 | A | A | B | A | B | A | A | A |
| ss_128625 | 182.32 | 45,581,205 | A | B | B | A | B | A | B | A |
| ss_128603 | 182.34 | 45,586,061 | B | B | A | B | A | B | B | A |
| ***qBD4*** | | | **H1** | **H2** | **H3** | **H4** | **H5** |  |  |  |
| ss_413934 | 47.83 | 11,956,738 | B | B | A | A | A |  |  |  |
| ss_414387 | 48.43 | 12,107,191 | B | A | B | B | B |  |  |  |
| ss_415301 | 50.09 | 12,523,245 | B | B | A | B | B |  |  |  |
| ss_417094 | 51.89 | 12,971,285 | B | B | A | B | A |  |  |  |
| ss_417310 | 52.14 | 13,034,674 | B | B | A | B | B |  |  |  |
| ss_417637 | 52.31 | 13,078,233 | B | B | A | B | A |  |  |  |
| ss_417666 | 52.37 | 13,091,850 | B | A | B | B | B |  |  |  |
| ss_417715 | 52.43 | 13,108,512 | A | A | B | A | A |  |  |  |
| ss_417840 | 52.49 | 13,123,061 | B | B | A | B | B |  |  |  |
| ss_418024 | 52.91 | 13,227,341 | A | B | A | A | B |  |  |  |
| ss_418108 | 53.09 | 13,271,987 | A | A | B | A | B |  |  |  |
| ss_418890 | 53.77 | 13,442,233 | A | B | A | A | A |  |  |  |
| ss_419614 | 54.54 | 13,633,831 | A | A | B | A | B |  |  |  |
| ***qBD7*** | | | **H1** | **H2** | **H3** | **H4** | **H5** | **H6** | **H7** |  |
| ss_778568 | 62.05 | 15,513,277 | B | B | A | A | A | B | A |  |
| ss_778587 | 62.14 | 15,535,610 | B | B | B | A | A | B | A |  |
| ss_778808 | 62.48 | 15,620,500 | A | A | A | B | B | A | B |  |
| ss_779224 | 62.87 | 15,717,070 | B | B | A | A | A | B | B |  |
| ss_779362 | 63.14 | 15,784,304 | B | B | A | B | A | A | B |  |
| ss_780816 | 65.46 | 16,365,104 | A | A | B | B | A | B | A |  |
| ss_781062 | 65.76 | 16,439,849 | A | A | A | B | A | B | A |  |
| ss_781249 | 66.04 | 16,511,121 | A | A | A | B | A | A | A |  |
| ss_781317 | 66.11 | 16,526,406 | A | A | B | B | A | B | A |  |

| Table S7. (*Cont.)* | | | | | | | | | | |
| --- | --- | --- | --- | --- | --- | --- | --- | --- | --- | --- |
| **SNP name** | **Genetic position (cM)** | **Physical location** | **Haplotype** | | | | | | | |
| ***qBD7*** *(cont.)* | | | **H1** | **H2** | **H3** | **H4** | **H5** | **H6** | **H7** |  |
| ss_781352 | 66.17 | 16,541,302 | B | B | B | A | B | A | B |  |
| ss_781455 | 66.27 | 16,567,648 | A | A | A | B | A | B | A |  |
| ss_782427 | 68.82 | 17,205,367 | A | B | B | A | B | B | B |  |
| snp_7_17628094 | 68.91 | 17,226,623 | B | A | B | B | A | A | A |  |
| ***qRD4* and *qFDP4*** | | | **H1** | **H2** | **H3** | **H4** |  |  |  |  |
| ss_409901 | 42.33 | 10,582,092 | A | B | B | A |  |  |  |  |
| ss_410134 | 42.51 | 10,626,874 | B | B | A | A |  |  |  |  |
| ss_410165 | 42.56 | 10,641,209 | B | B | A | A |  |  |  |  |
| ss_410336 | 42.70 | 10,676,008 | B | B | A | B |  |  |  |  |
| ss_410398 | 42.79 | 10,696,489 | B | B | A | A |  |  |  |  |
| ss_410478 | 43.04 | 10,760,086 | B | B | A | B |  |  |  |  |
| ss_410794 | 43.56 | 10,890,653 | B | B | A | A |  |  |  |  |
| ss_410955 | 43.62 | 10,904,526 | B | B | A | A |  |  |  |  |
| ss_411147 | 43.69 | 10,921,604 | B | B | A | B |  |  |  |  |
| ss_411188 | 43.69 | 10,923,251 | A | A | B | A |  |  |  |  |
| ss_411196 | 43.69 | 10,923,464 | B | B | A | A |  |  |  |  |
| ss_411601 | 43.91 | 10,976,364 | B | B | A | B |  |  |  |  |
| ss_411637 | 43.93 | 10,981,971 | B | B | A | B |  |  |  |  |
| ss_412338 | 44.83 | 11,208,347 | B | B | A | B |  |  |  |  |
| ss_412662 | 45.19 | 11,298,736 | A | A | B | B |  |  |  |  |
