# Supplementary material for: Mapping and characterization QTLs for phenological traits in seven pedigree-connected peach families
Source: BMC Genomics. 2021 Mar 16;22:187. doi: 10.1186/s12864-021-07483-8 (PMC7962356; doi:10.1186/s12864-021-07483-8)
Supplement: Supplementary file 2 — Additional file 2: Supplemental Figure S1-S5. [file 12864_2021_7483_MOESM2_ESM.docx]

| 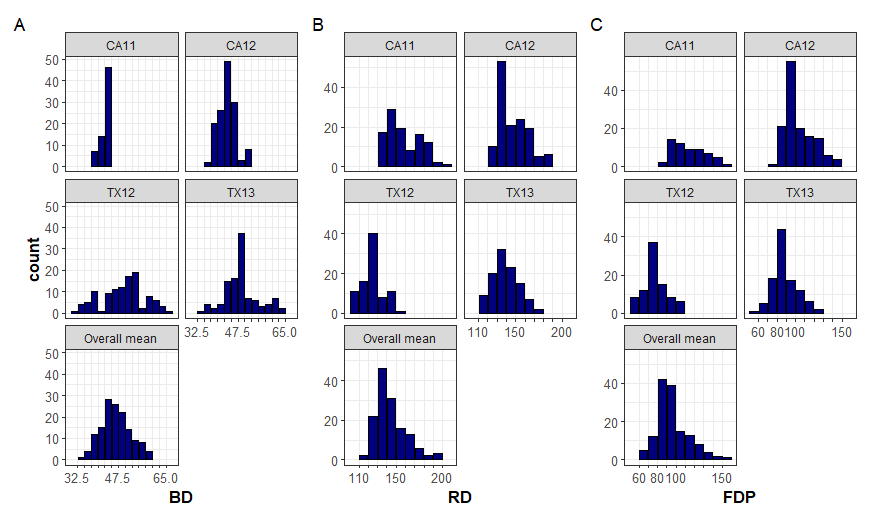 |
| --- |
| Fig. S1. Histograms for bloom date (BD) (A), ripening date (RD) (B), and fruit development period (FDP) (C) of seven full-sib peach families evaluated in different environments and the overall combined mean.  CA11, CA12 = Fowler, California 2011 and 2012; TX12 = College Station, Texas 2012; Overall mean = mean across environments. |

| 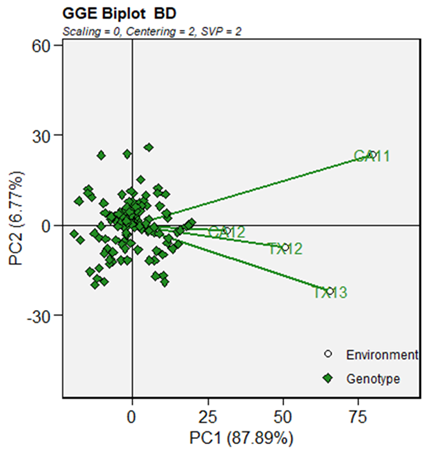 | 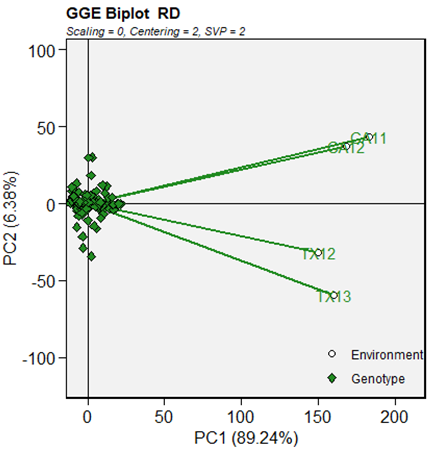 |
| --- | --- |
| 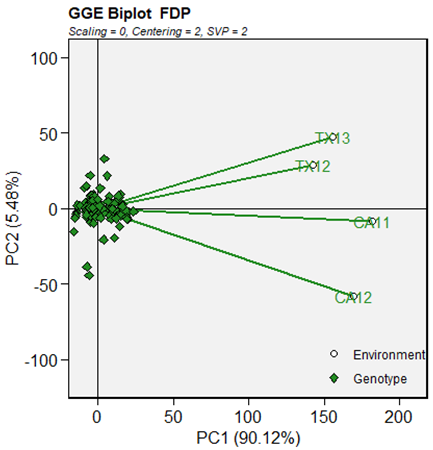 |  |
| Fig. S2. GGE biplots for bloom date (BD), ripening date (RD), and fruit development period (FDP) of seven full-sib peach families showing the relationship between Fowler, CA in 2011 and 2012 and College Station, TX in 2012, and 2013 environments.  TA was not evaluated in TX 2013. | |

| BD-CA11 | |
| --- | --- |
| 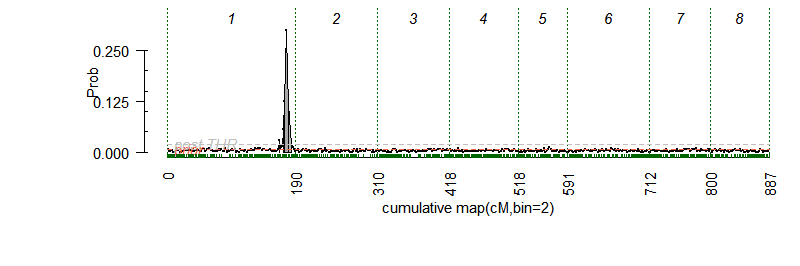 | 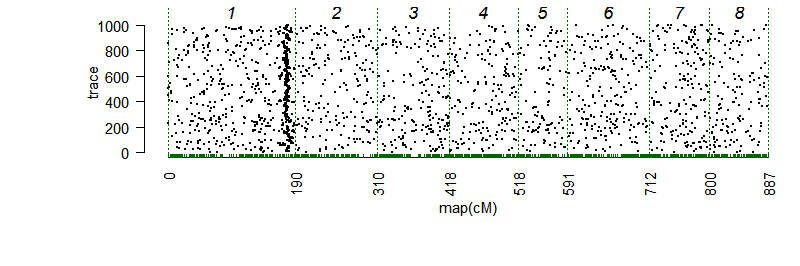 |
| BD-CA12 | |
| 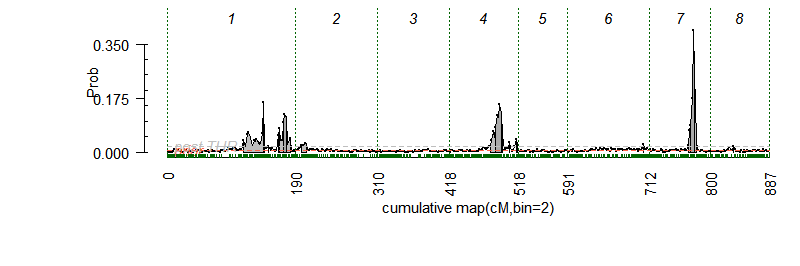 | 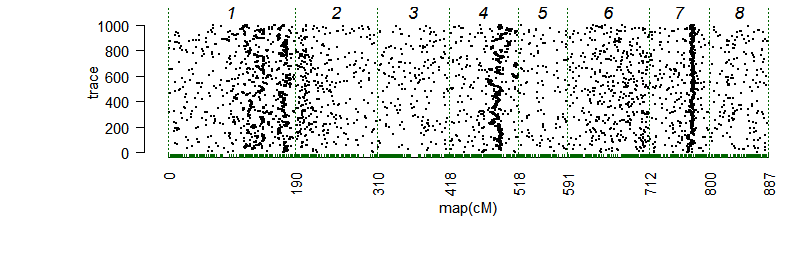 |
| BD-TX12 | |
| 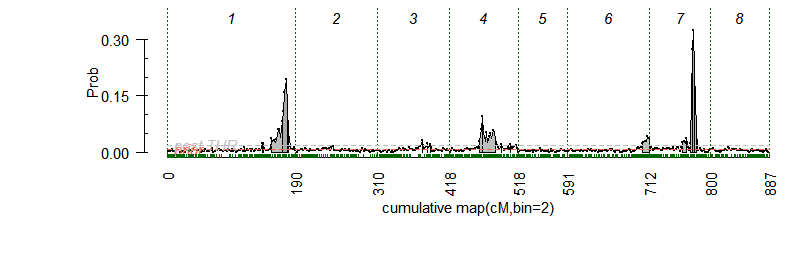 | 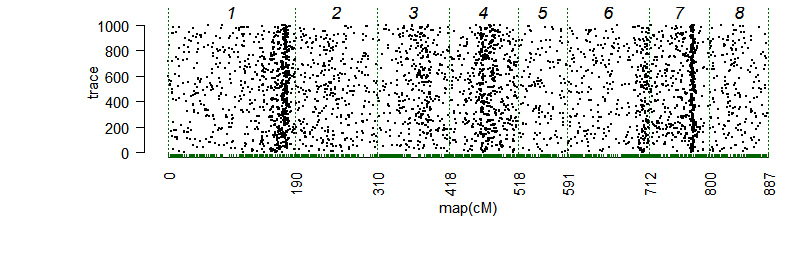 |
| BD-TX13 | |
| 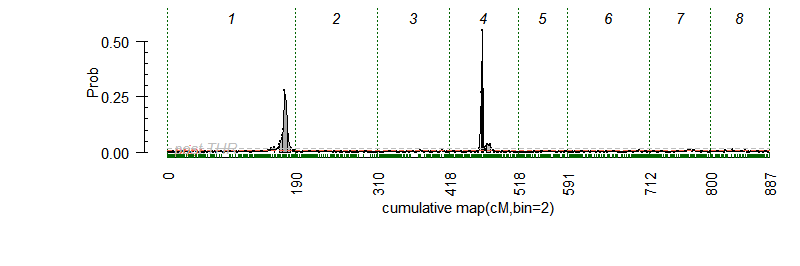 | 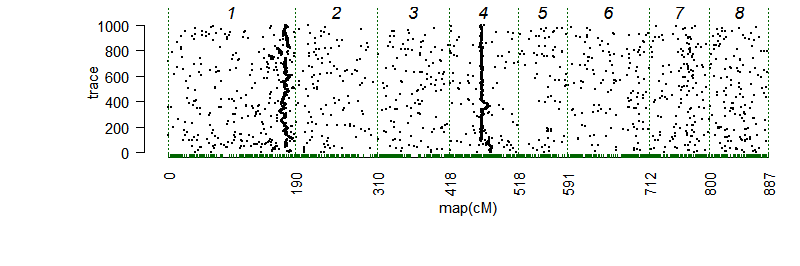 |
| BD-mean | |
| 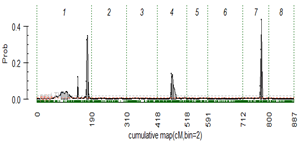 | 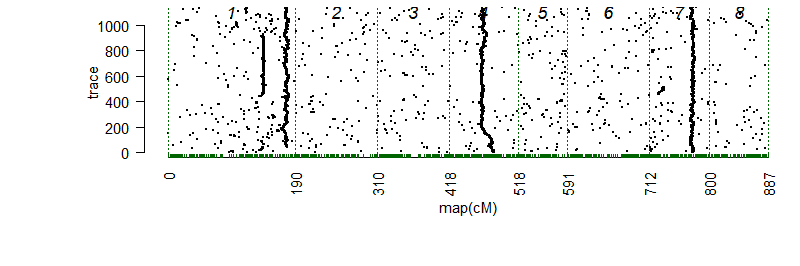 |
| Fig. S3. Posterior positions (left) and trace samples QTL positions (right) based on an additive model performed using Visual FlexQTL software (Bink, 2008) for the bloom date (BD) from four environments (CA11, CA12, TX12, TX13), and the overall combined mean for 143 peach seedlings.  CA11, CA12 = Fowler, California 2011 and 2012; TX12, TX13 = College Station, Texas 2012 and 2013. | |

| RD-CA11 | |
| --- | --- |
| 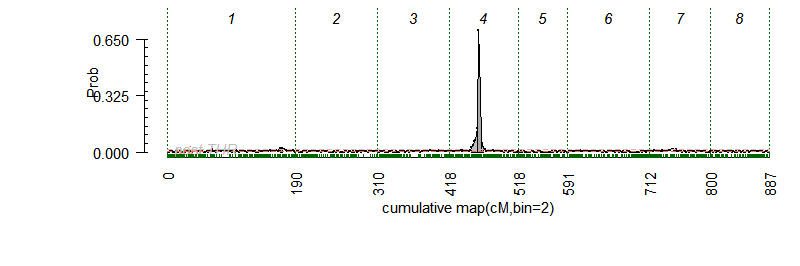 | 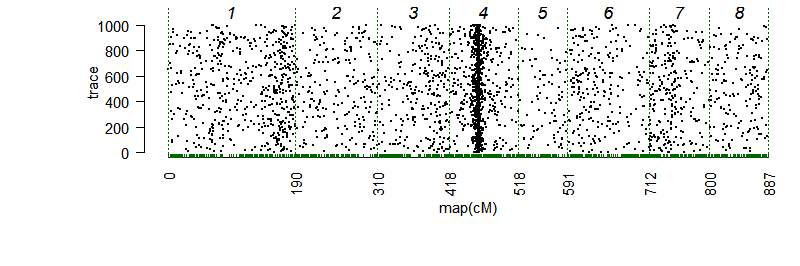 |
| RD-CA12 | |
| 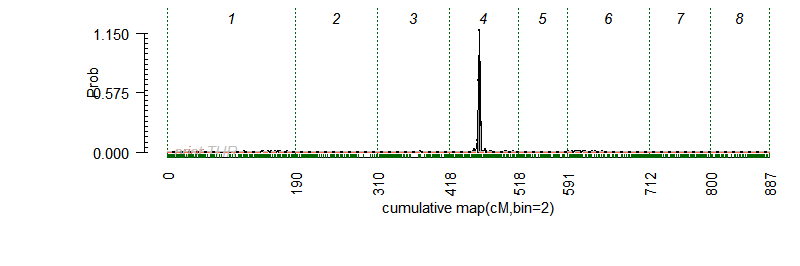 | 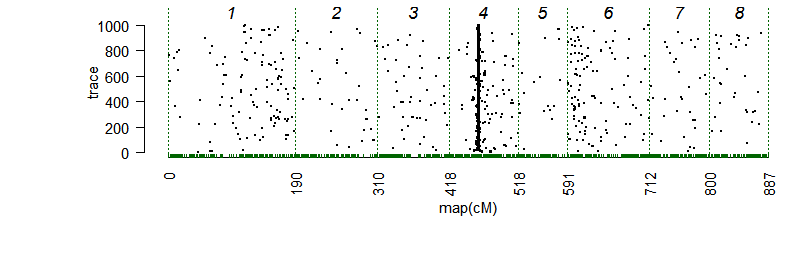 |
| RD-TX12 | |
| 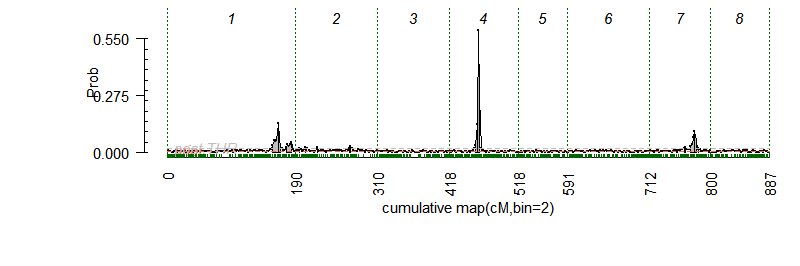 | 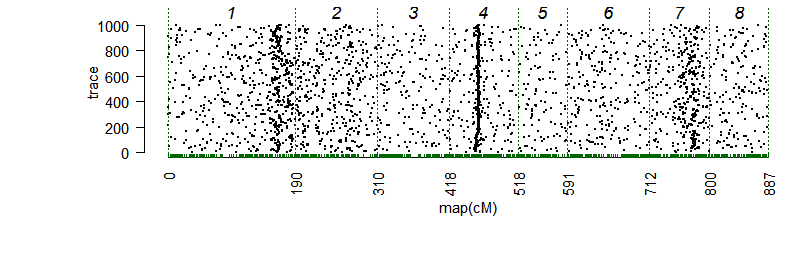 |
| RD-TX13 | |
| 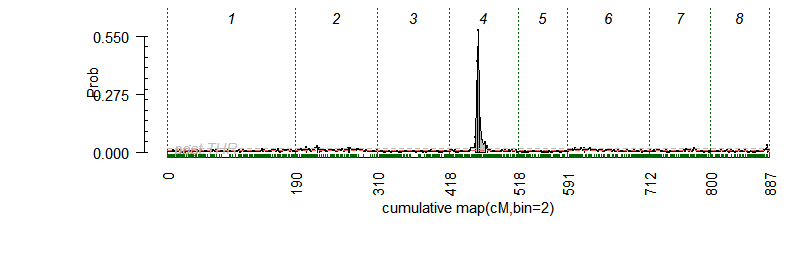 | 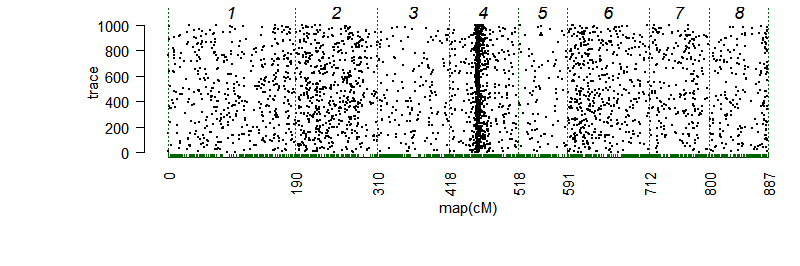 |
| RD-mean | |
| 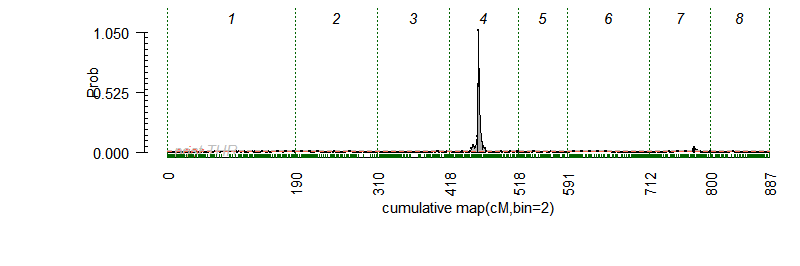 | 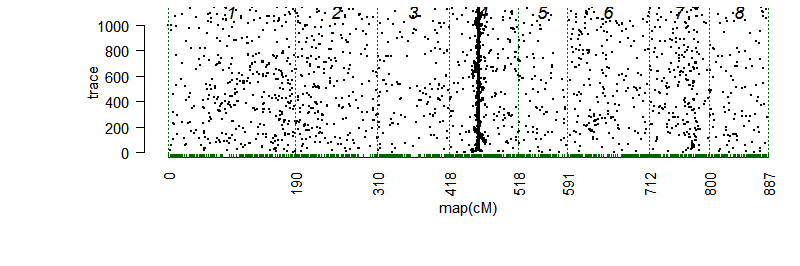 |
| Fig. S4. Posterior positions (left) and trace samples QTL positions (right) based on an additive model performed using Visual FlexQTL software (Bink, 2008) for the ripening date (RD) from four environments (CA11, CA12, TX12, TX13), and the overall combined mean for 143 peach seedlings.  CA11, CA12 = Fowler, California 2011 and 2012; TX12, TX13 = College Station, Texas 2012 and 2013. | |

| FDP-CA11 | |
| --- | --- |
| 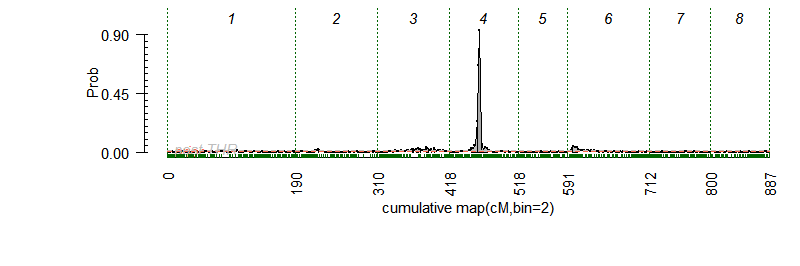 | 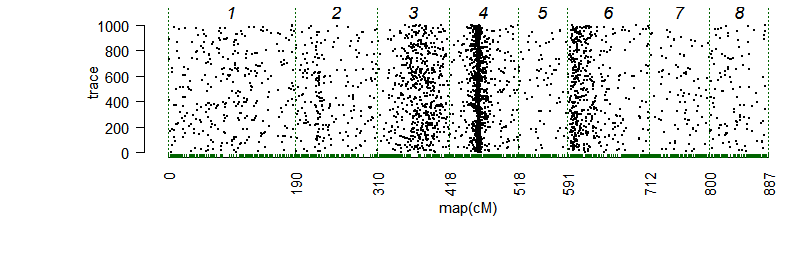 |
| FDP-CA12 | |
| 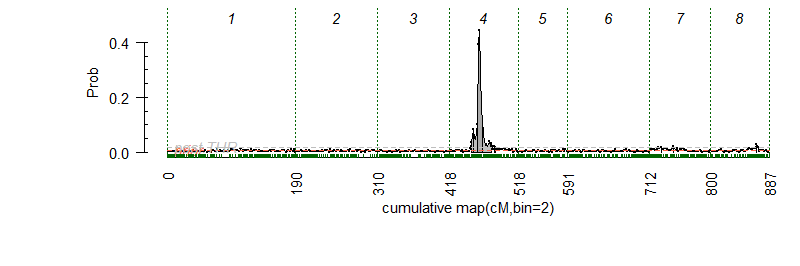 | 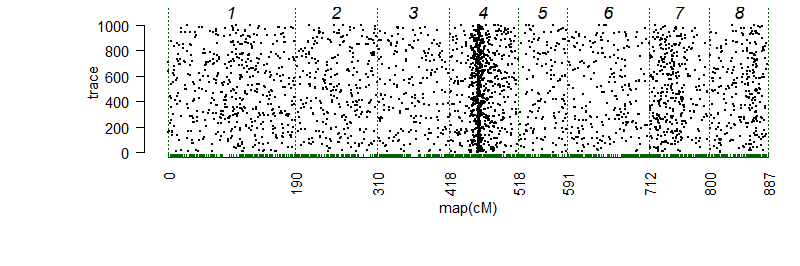 |
| FDP-TX12 | |
| 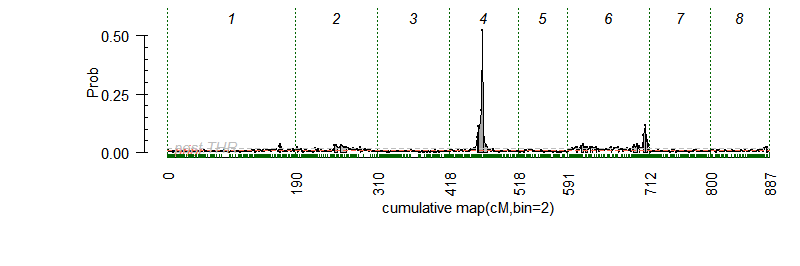 | 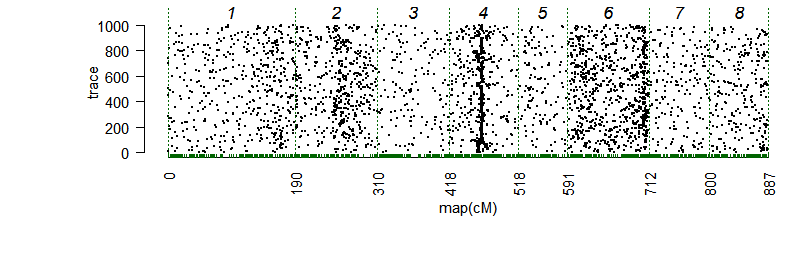 |
| FDP-TX13 | |
| 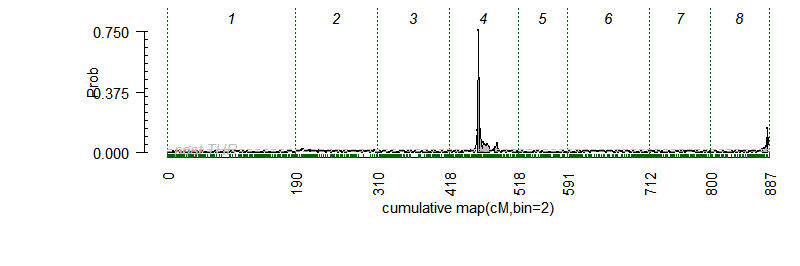 | 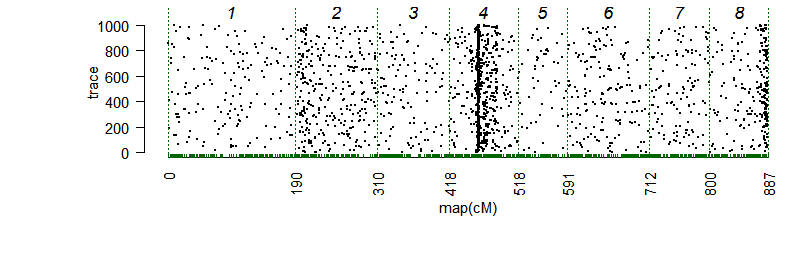 |
| FDP-mean | |
| 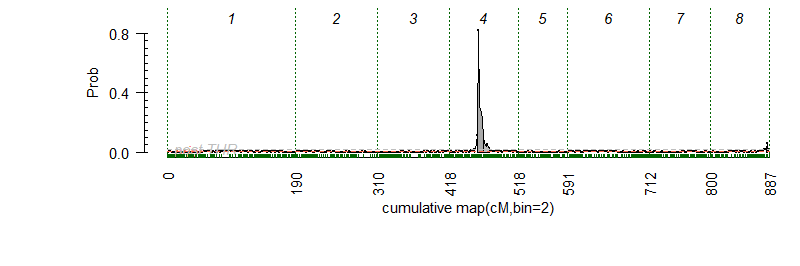 | 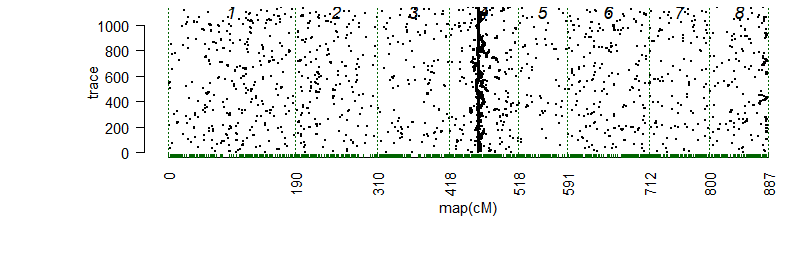 |
| Fig. S5. Posterior positions (left) and trace samples QTL positions (right) based on an additive model performed using Visual FlexQTL software (Bink, 2008) for the fruit development period (FDP) from four environments (CA11, CA12, TX12, TX13), and the overall combined mean for 143 peach seedlings.  CA11, CA12 = Fowler, California 2011 and 2012; TX12, TX13 = College Station, Texas 2012 and 2013. | |
